# Mammalian RNase H1 directs RNA primer formation for mtDNA replication initiation and is also necessary for mtDNA replication completion

**DOI:** 10.1093/nar/gkac661

**Published:** 2022-08-10

**Authors:** Jelena Misic, Dusanka Milenkovic, Ali Al-Behadili, Xie Xie, Min Jiang, Shan Jiang, Roberta Filograna, Camilla Koolmeister, Stefan J Siira, Louise Jenninger, Aleksandra Filipovska, Anders R Clausen, Leonardo Caporali, Maria Lucia Valentino, Chiara La Morgia, Valerio Carelli, Thomas J Nicholls, Anna Wredenberg, Maria Falkenberg, Nils-Göran Larsson

**Affiliations:** Department of Medical Biochemistry and Biophysics, Karolinska Institutet, Stockholm 17177, Sweden; Max Planck Institute for Biology of Ageing, 50931 Cologne, Germany; Department of Medical Biochemistry and Cell Biology, University of Gothenburg, PO Box 440, Gothenburg 405 30, Sweden; Department of Medical Biochemistry and Cell Biology, University of Gothenburg, PO Box 440, Gothenburg 405 30, Sweden; Key Laboratory of Growth Regulation and Translational Research of Zhejiang Province, School of Life Sciences, Westlake University, Hangzhou, Zhejiang 310024, China; Department of Medical Biochemistry and Biophysics, Karolinska Institutet, Stockholm 17177, Sweden; Department of Medical Biochemistry and Biophysics, Karolinska Institutet, Stockholm 17177, Sweden; Department of Medical Biochemistry and Biophysics, Karolinska Institutet, Stockholm 17177, Sweden; Harry Perkins Institute of Medical Research and ARC Centre of Excellence in Synthetic Biology, Nedlands, WA 6009, Australia; Department of Medical Biochemistry and Cell Biology, University of Gothenburg, PO Box 440, Gothenburg 405 30, Sweden; Harry Perkins Institute of Medical Research and ARC Centre of Excellence in Synthetic Biology, Nedlands, WA 6009, Australia; Telethon Kids Institute, Northern Entrance, Perth Children's Hospital, 15 Hospital Avenue, Nedlands, WA, Australia; Department of Medical Biochemistry and Cell Biology, University of Gothenburg, PO Box 440, Gothenburg 405 30, Sweden; IRCCS Istituto delle Scienze Neurologiche di Bologna, Programma di Neurogenetica, Bologna, Italy; IRCCS Istituto delle Scienze Neurologiche di Bologna, Programma di Neurogenetica, Bologna, Italy; Department of Biomedical and Neuromotor Sciences (DIBINEM), University of Bologna, Bologna, Italy; IRCCS Istituto delle Scienze Neurologiche di Bologna, Programma di Neurogenetica, Bologna, Italy; IRCCS Istituto delle Scienze Neurologiche di Bologna, Programma di Neurogenetica, Bologna, Italy; Department of Biomedical and Neuromotor Sciences (DIBINEM), University of Bologna, Bologna, Italy; Wellcome Centre for Mitochondrial Research, Biosciences Institute, Newcastle University, Framlington Place, Newcastle upon Tyne NE2 4HH, UK; Department of Medical Biochemistry and Biophysics, Karolinska Institutet, Stockholm 17177, Sweden; Centre for Inherited Metabolic Diseases, Karolinska University Hospital, Stockholm, Sweden; Department of Medical Biochemistry and Cell Biology, University of Gothenburg, PO Box 440, Gothenburg 405 30, Sweden; Department of Medical Biochemistry and Biophysics, Karolinska Institutet, Stockholm 17177, Sweden

## Abstract

The *in vivo* role for RNase H1 in mammalian mitochondria has been much debated. Loss of RNase H1 is embryonic lethal and to further study its role in mtDNA expression we characterized a conditional knockout of *Rnaseh1* in mouse heart. We report that RNase H1 is essential for processing of RNA primers to allow site-specific initiation of mtDNA replication. Without RNase H1, the RNA:DNA hybrids at the replication origins are not processed and mtDNA replication is initiated at non-canonical sites and becomes impaired. Importantly, RNase H1 is also needed for replication completion and in its absence linear deleted mtDNA molecules extending between the two origins of mtDNA replication are formed accompanied by mtDNA depletion. The steady-state levels of mitochondrial transcripts follow the levels of mtDNA, and RNA processing is not altered in the absence of RNase H1. Finally, we report the first patient with a homozygous pathogenic mutation in the hybrid-binding domain of RNase H1 causing impaired mtDNA replication. In contrast to catalytically inactive variants of RNase H1, this mutant version has enhanced enzyme activity but shows impaired primer formation. This finding shows that the RNase H1 activity must be strictly controlled to allow proper regulation of mtDNA replication.

## INTRODUCTION

The ribonuclease H (RNase H) family of enzymes are endonucleases that cleave the RNA component of RNA:DNA hybrids without sequence specificity, and are found in prokaryotes, archaea and eukaryotes (1). Two main groups of this endonuclease family, RNase H1 and RNase H2, are found in all three domains of life and are the only variants present in mammalian cells. The mammalian RNase H2 is an obligate heterotrimer consisting of a catalytic subunit, RNase H2A, complexed with two structural subunits, RNase H2B and RNase H2C. The RNase H2 enzyme is only present in the nucleus and has been reported to be important for removal of mis-incorporated ribonucleotides and RNA:DNA hybrids formed during DNA replication and repair (2–4). In contrast, RNase H1 is a monomeric enzyme that has been reported to have two isoforms created by alternate translation start sites in the encoding mRNA (5). The longer isoform contains a mitochondrial targeting sequence (MTS) directing import of RNase H1 into mitochondria, whereas the predicted shorter isoform lacks the MTS and is localized to the nucleus. In the nucleus, RNase H1 has been suggested to contribute to genome stability and to preserve telomere integrity by resolving R-loops (6–8), which are triple-stranded structures comprised of an RNA:DNA hybrid and a displaced DNA strand. It is well known that R-loops arise from different sources and can be resolved by several enzymatic activities in the nucleus such as topoisomerases and helicases (9–12). RNase H1 has been implicated in the removal of R-loops formed during nuclear DNA replication (6), immunoglobulin class switch DNA recombination (13) or as by-products of rDNA transcription in the nucleoli (14). In mitochondria, RNase H1 is essential for mitochondrial DNA (mtDNA) replication (15) and has been linked to several aspects of the expression and replication of mtDNA. In the mouse, homozygous *Rnaseh1* knockout causes severe mtDNA depletion and developmental arrest at embryonic day (E) 8.5 (15). A study of cultured mouse embryonic fibroblasts (MEFs) lacking RNase H1 reported that it is required for primer removal during mtDNA replication (16). In support of this model, an *in vitro* study of mtDNA replication initiation at the light-strand origin of replication (O_L_) with recombinant replication factors revealed an important role for RNase H1 in RNA primer removal (17). However, RNase H1 can only partly remove primers as it leaves 1–3 ribonucleotides at the RNA-DNA junction (17), consistent with its known biochemical properties (18). A subsequent study of patient cell lines expressing mutant RNase H1 combined with *in vitro* biochemistry suggested that RNase H1 also has a role in primer maturation by generating the 3′ RNA-ends that the mitochondrial DNA polymerase γ (POLγ) uses for replication initiation (19). In addition to these two proposed roles in mtDNA replication, i.e. primer maturation and removal, RNase H1 has also been reported to regulate transcription of 7S RNA, an abundant non-coding mtDNA transcript linked to replication primer synthesis (20). Furthermore, RNase H1 has been suggested to play a role in pre-rRNA processing via interaction with the P32 protein of the mitochondrial nucleoid (21). Constitutive and inducible knockout of RNase H1 in mouse liver causes severe liver degeneration due to mitochondrial dysfunction suggested to be caused by R-loop accumulation resulting in reduced transcription and replication of mtDNA (22). Although the exact role of RNase H1 in mammalian mitochondria *in vivo* needs further clarification, additional support for a critical role in mtDNA maintenance comes from studies of patients with mutations in *RNASEH1* (23–26). A range of pathogenic mutations have been found in *RNASEH1* that directly reduces the catalytic function of the enzyme. Affected patients display typical adult-onset mitochondrial disease phenotypes, such as mitochondrial myopathy with exercise intolerance, muscle weakness with progressive external ophthalmoplegia (PEO) and ptosis, and central nervous system involvement with cerebellar atrophy and ataxia (23–26). In these patients, genetic analysis of skeletal muscle biopsy specimens typically demonstrates rearrangements often accompanied by depletion of mtDNA (23–26).

In this study, we have extensively characterized the *in vivo* role of mouse RNase H1 and report that it is essential both during embryonic development and for the function of differentiated organs, such as the heart and skeletal muscle. Loss of RNase H1 in the heart leads to mtDNA replication stalling with accumulation of linear deleted mtDNA fragments and mtDNA depletion. Ablation of RNase H1 also leads to low levels of mtDNA-encoded transcripts and deficient oxidative phosphorylation (OXPHOS), which is likely related to the marked mtDNA depletion as no transcription elongation or RNA processing defects are observed. We provide evidence that RNase H1 is necessary to restrict RNA replication primer formation to specific sites at the heavy-strand origin of replication (O_H_) and O_L_ in conditional knockout hearts of the mouse. Finally, we report a mitochondrial disease patient with altered mtDNA replication carrying a novel homozygous pathogenic mutation that makes RNase H1 more active in processing RNA:DNA hybrids than the wild-type enzyme. These findings demonstrate that the RNase H1 activity needs to be carefully balanced to promote accurate mtDNA replication.

## MATERIALS AND METHODS

### Mouse models

*Rnaseh1* knockout mice were generated by Taconic Artemis by targeting the gene in embryonic stem cells derived from C57BL/6N using BAC clones from C57BL/6J RPCI-23 BAC library. To generate the *Rnaseh1* knockout allele, exon II of the *Rnaseh1* locus was flanked by loxP sites. The puromycin resistance cassette was introduced as a selection marker and removed by mating *Rnaseh1^+/loxP-pur^* mice with transgenic mice ubiquitously expressing Flp recombinase. To obtain germline knockout mice, *Rnaseh1^+/loxP^* mice were mated with mice ubiquitously expressing Cre recombinase under the β-actin promotor to generate heterozygous knockout *Rnaseh1^+/−^* mice. The tissue-specific conditional knockout of RNase H1 in heart and skeletal muscle was obtained by crossing *Rnaseh1^+/loxP^* mice with transgenic mice expressing Cre recombinase under the control of the muscle creatinine kinase promoter (*Ckmm-cre*). Animals were housed in a 12-h light/dark cycle at 21°C and fed with a normal chow diet *ad libitum*. Animal studies were approved by the animal welfare ethics committee and performed in compliance with National and European law. Mice at the age of 8, 16 and 24 weeks were used for biochemical analyses.

### Cell lines

Human embryonic kidney cells (HEK293T) and patient-derived fibroblast lines were used for *in vitro* studies. Cells were cultured in DMEM GlutaMax (Invitrogen) medium supplemented with 10% fetal bovine serum (FBS) and 1× PenStrep and grown in an incubator at 37°C and 5% CO_2_. The patient fibroblast line carrying a homozygous c.424G > A (V142I) mutation in the H-domain of RNase H1 was obtained from a patient diagnosed at Karolinska University Hospital. This patient presented with PEO and muscular fatigue. A muscle biopsy specimen from the *tibialis anterior* muscle at 41 years of age showed a general reduction in the mitochondrial ATP production rate and a combined respiratory enzyme complex deficiency. Histochemical analysis revealed numerous ragged-red and COX-negative muscle fibers. This mutation has been previously reported (23). Control fibroblast lines C1-3 were obtained from healthy individuals at Karolinska University Hospital. The patient fibroblast line carrying a novel homozygous c.86A > G (Y29C) mutation in the HBD of RNase H1 was obtained from a patient diagnosed at the IRCCS Istituto delle Scienze Neurologiche di Bologna in Italy. The male proband and a sister had an identical phenotype of adult-onset PEO and ptosis, associated with peripheral neuropathy, sensorineural deafness and mitochondrial myopathy with abundant COX-negative fibers. Later in life, both siblings developed parkinsonism and eventually cognitive deterioration, and died in their 80s. In addition, we studied a patient fibroblast line with a novel loss-of-function mutation in *MGME1*, c.563_564delCAinsT (S188YfsX7). This patient was diagnosed at the IRCCS Istituto delle Scienze Neurologiche di Bologna in Italy and had early-onset PEO and ptosis, with mild mitochondrial myopathy and MRI of the brain showed evidence of cerebellar atrophy. The control fibroblast lines, C4 and C5, were obtained from healthy individuals at IRCCS Istituto delle Scienze Neurologiche di Bologna in Italy. For all individuals recruited in Italy, informed consent was obtained for fibroblast cell line establishment and the study was approved by the institutional ethical board (Comitato Etico Interaziendale Bologna-Imola, #CE-BI 13036).

### Isolation of mitochondria from mouse tissue

Mitochondria were isolated from mouse heart using differential centrifugation as previously described (27). Following dissection, fresh tissues were cut and washed with ice cold PBS and homogenized in mitochondrial isolation buffer consisting of 310 mM sucrose, 10 mM Tris–HCl, 1 mM EDTA and freshly added 1× Complete protease inhibitor cocktail (Roche). The homogenate was centrifuged at 1000 *g* for 10 min at 4°C to remove the cell debris and nuclei. The supernatant was collected and spun at 10 000 *g* for 15 min at 4°C to isolate mitochondria. Crude mitochondrial pellets were resuspended in mitochondria isolation buffer and protein concentration was determined with the Qubit 4.0 fluorometer (Invitrogen).

### Isolation of mitochondria from cultured cells

Mitochondria were isolated from cultured cells harvested after reaching 80–90% confluency. Cells were washed two times in cold PBS. The cell pellet was resuspended in 8 ml of mitochondria isolation buffer consisting of 20 mM HEPES pH 7.6, 220 mM mannitol, 70 mM sucrose, 1 mM EDTA and freshly added 1× Complete protease inhibitor cocktail (Roche) and 2 mg/ml of fatty acid-free bovine serum albumin (BSA). To facilitate swelling, the cell suspension was incubated on ice for 30 min. Subsequently, homogenization was performed with 40 strokes by using a manual homogenizer. The cell homogenate was centrifuged at 800 *g* for 10 min at 4°C to remove the cell debris. The supernatant was collected and centrifuged at 10 000 *g* for 10 min at 4°C. The mitochondrial pellet was collected and resuspended in mitochondria isolation buffer without BSA and centrifuged at 10 000 for 10 min at 4°C. Protein quantification was performed with the Qubit 4.0 fluorometer (Invitrogen).

### Cellular fractionation

HEK293T cells were cultured until reaching 80–90% confluency. Cells were then harvested, pelleted by centrifugation at 800 *g* for 7 min at 4°C and washed two times in cold PBS. Mitochondria-, cytosol- and nuclei-containing fractions were obtained by using the Abcam Cell Fractionation Kit according to the instructions. The fractions were further analyzed by SDS-PAGE and western blots. The antibodies used for this experiment are listed in Supplementary Table S1.

### Protease protection assay

For each treatment, 1 mg of isolated mitochondria (see isolation of mitochondria from cultured cells**)** from HEK293T cells were pelleted, resuspended and incubated for 30 min in 400 μl mitochondria isolation buffer [20 mM HEPES pH 7.6, 220 mM mannitol, 70 mM sucrose, 1 mM EDTA with freshly added 1× Complete protease inhibitor cocktail (Roche) and 2 mg/ml of fatty acid-free bovine serum albumin (BSA)], hypotonic swelling buffer (10 mM HEPES–KOH, pH 7.4) or mitochondria isolation buffer supplemented with 1% Triton X-100. Each of the three aliquots of mitochondria in different buffers were further subdivided into two aliquots that contained proteinase K at a final concentration of 60 μg/ml or no proteinase K. After 20 min of incubation on ice, protease K activity was inhibited with 2 μl of 0.1 M phenylmethylsulfonyl fluoride (PMSF) added to each sample. Proteins were precipitated with 0.02% sodium deoxycholate and 12% trichloroacetic acid. Finally, samples were analyzed by SDS-PAGE and western blots. The antibodies used for this experiment are listed in Supplementary Table S1.

### DNA extraction and mtDNA quantification

Genomic DNA from mouse tissues or human cells was isolated using the Gentra Puregene Tissue Kit (Qiagen) according to instructions. Total DNA from E8.5 embryos was isolated by incubation in 24 mM NaOH, 0.2 mM EDTA at 96°C for 40 min. The extracts were neutralized by adding 40 mM Tris (pH 7.8). DNA quantification was performed with the Qubit 4.0 fluorometer (Invitrogen). The mtDNA copy number was measured by qPCR in a QuantStudio 6 Flex Real-Time PCR System using the TaqMan™ Universal Master Mix II with UNG and TaqMan probes for *Nd5*, *Atp6* and *16S*. The *18S* probe was used to normalize samples to the nuclear DNA content. The mtDNA copy number in patient muscle biopsies was quantified by qPCR as previously described (28). The mtDNA deletions in patient muscle biopsies were quantified by droplet digital PCR (ddPCR) with specific probes in *MT-ND4* and *MT-ND1* (29). The 7S DNA in patient muscle biopsies was quantified by ddPCR by amplifying two mtDNA regions, one within and one outside of the 7S DNA region (29).

### Southern blot analyses

Murine genomic DNA (or mtDNA where indicated), 2 μg (or more where indicated), was digested with one of the following enzymes: SacI, XhoI, BglII or EagI. Human genomic DNA was digested with the EcoRI and BamHI enzymes. Linearized DNA fragments were separated in 0.8% agarose gels and transferred to Hybond-N+ membranes (GE Healthcare). To release 7S DNA, samples were heated for 3 min at 93°C prior to loading. Next, membranes were hybridized with α-[^32^P]-dCTP-labelled dsDNA probes to detect total mtDNA (pAM1 probe for mouse, and CytB for human samples), 7S DNA, or nuclear 18S rDNA as a loading control. Radioactive signals were visualized using PhosphorImager screens and a Typhoon 7000 FLA (GE Healthcare).

### *In organello* replication assay

Freshly isolated mitochondria (1 mg) were washed two times in 0.5 ml of cold incubation buffer [25 mM sucrose, 75 mM sorbitol, 100 mM KCl, 10 mM K_2_HPO_4_, 0.05 mM EDTA, 5 mM MgCl_2_, 10 mM glutamate, 2.5 mM malate, 10 mM Tris–HCl pH (7.4)] supplemented with fatty acid-free BSA (1 mg/ml) and 1 mM ADP. Mitochondria were then pelleted at 8000 *g* for 4 min and resuspended in 0.5 ml of prewarmed incubation buffer supplemented with 1 mg/ml of fatty acid-free BSA (1 mg/ml), 1 mM ADP, 50 μM each of dTTP, dCTP and dGTP and 20 μCi α-[^32^P]-dATP. Next, samples were incubated for 2 h at 37°C on a rotating wheel. After incubation mitochondria were pelleted at 8000 *g* for 4 min and washed three times with washing buffer [10% glycerol, 0.15 mM MgCl_2_, 10 mM Tris–HCl pH (6,8)]. mtDNA was extracted with a Gentra Puregene Tissue Kit, digested with Sac I HF enzyme, separated on a 0.8% agarose gel and transferred to Hybond-N+ membrane. The signal from the membrane was visualized by autoradiography. To control for mitochondrial amount used in the assay, a protein sample, taken before the mtDNA extraction, was resolved by SDS-PAGE and transferred to a polyvinylidene difluoride (PVDF) membrane, which was incubated with an antibody against voltage-dependent anion channel (VDAC).

### 2DNAGE

Fresh heart mitochondria were isolated and mtDNA was extracted using the Gentra Puregene Tissue Kit without RNase A treatment. The mtDNA was digested with BclI, precipitated and 3 μg from each sample was loaded onto the first-dimension gels (0.4% agarose without ethidium bromide). Electrophoresis was carried out at 27 V for 18 h at room temperature. The lanes containing DNA were excised, turned 90° counterclockwise and cast with 1% agarose (500 ng/ml ethidium bromide). Second dimension gels were separated at constant 260 mA for 6 h at 4°C. Gels were transferred to nitrocellulose membranes by Southern blotting, and probed with PCR-generated DNA fragments detecting either the O_H_-containing (nt 12 034–16 180) or the O_L_-containing (nt 3102–7084) region.

### Primer extension

Total DNA was isolated from the mouse heart tissue with the Gentra Puregene Tissue Kit without RNase A treatment. An 3 μg aliquot of DNA was incubated for 1 h at 37°C with or without RNase H2 (NEB). Primer extension was performed with 2 U Taq DNA polymerase (NEB) in 1× ThermoPol buffer, 200 μM dNTPs and 1.5 pmol labelled primer. The primer, corresponding to nt 15 181–15 201, was 5′-end labelled with γ-[^32^P]-ATP using PNK (NEB). The primer extension reaction was initiated for 5 min at 95°C and then continued for 25 cycles with 30 s at 95°C, 30 s at 58°C, 45 s at 72°C and 5 min at 72°C. The reactions were terminated and precipitated with ethanol.

### Analysis of mtDNA deletions using deep sequencing

Total or mitochondrial DNA was extracted from mouse heart or brain, libraries were prepared using TruSeq Nano DNA Library Preparation Kit, and sequenced using NovaSeq 6000. Analyses were performed following a previously described pipeline (30). To avoid false negatives at Nuclear Mitochondrial Segments (NUMTs), reads were directly aligned to the mouse mitochondrial genome (GRCm38) to identify deletions and duplications, and then gapped alignments, indicative of deletions/duplications, were clustered and visualized. Heteroplasmy levels were calculated by comparing the number of reads supporting the corresponding breakpoints to the total number of reads overlapping the breakpoints after removal of PCR duplicates.

### HydEn-sequencing

Free 5′ DNA ends of mtDNA were mapped by HydEn sequencing as previously described (31). Briefly, genomic DNA was isolated from mouse heart tissue with the Gentra Puregene Tissue Kit without RNase A treatment. HydEn-seq was performed by hydrolysis of 1 μg of genomic DNA with 0.3 M KOH for 2 h at 55°C. After hydrolysis, DNA was denatured for 3 min at 85°C, cooled on ice and treated with 10 U T4 Polynucleotide Kinase (3′ phosphatase minus) in 1 mM ATP for 20 min at 37°C and then treated for 20 min at 65°C. DNA was purified using 1.8 volumes of CleanNGS beads and eluted in 14 μl EB. A 13 μl aliquot of DNA was denatured for 3 min at 85°C and ligated to 1 μM ARC140 using 10 U T4 RNA ligase in 25% PEG3350, 1 mM CoCl_3_(NH3)_6_ and 20 μM ATP overnight at room temperature. The following day, two washes were performed using 0.8 volumes of CleanNGS beads and eluted with 20 μl EB in the first step and 13.8 μl EB in the second step. DNA was denatured for 3 min at 85°C and incubated for 5 min with 1xBF for T7 DNA polymerase, 0.2 mM dNTPs, 40 μg/ml BSA and 1 μM of the adapter consisting of ARC76/ARC77. Reactions were then treated with 4 U of T7 DNA polymerase for 5 min. Products were purified with 0.8 volumes of CleanNGS beads and amplified using primers ARC78–ARC84 and ARC160. All oligos are from IDT. Sequences are provided in Supplementary Table S1. The star (*) indicates a phosphorothioate bond. ARC140 contains a 5′-amino group instead of a 5′-OH group, in combination with a C6 linker. Purified libraries were then sequenced using the Illumina NextSeq500 instrument for 2 × 75 paired end reads. Reads were trimmed for quality and adapter sequence with cutadapt (-m 15 -q 10–match-read-wildcards). Pairs with one or both reads shorter than 15 nts were discarded. Mate 1 of the remaining pairs was aligned to an index containing the sequence of all oligos used in the preparation of these libraries with bowtie sing bowtie 0.12.8 (-m1 -v2), and all pairs with successful alignments were discarded. Pairs passing this filter were subsequently aligned to the mm10 *M. musculus* reference mitochondrial genome. Single-end alignments were then performed using mate 1 of all unaligned pairs (-v2). The count of ends of all paired-end and single-end alignments were determined and these counts were converted to bedGraph format for visualization.

### RT-qPCR and northern blot analyses

Total RNA from mouse heart was extracted using the TRIzol/chloroform extraction method. RNA was quantified with the Qubit 4.0 fluorometer (Invitrogen). RT-qPCR was used to determine *Rnaseh1* and mitochondrial mRNA levels and northern blotting was used to assess 7S RNA levels. For the qPCR analysis, the RNA samples were treated with deoxyribonuclease (DNase) to eliminate potential DNA contamination. Next, the cDNA synthesis was performed with High-Capacity cDNA Reverse Transcription Kit (Thermo Fisher) according to kit instructions. The qPCR was run in a QuantStudio 6 Flex Real-Time PCR System using TaqMan^TM^ Universal Master Mix II, with UNG and Taqman probes, *Rnaseh1*, *12S, 16S, Atp6, Atp8, Cox1, Cox2, Cox3, CytB, Nd1, Nd2, Nd3, Nd4, Nd5, Nd6*. *ActinB* was used for normalization. For northern blotting, total RNA (3 μg) was separated on a neutral 10% polyacrylamide gel. Next, separated RNAs were transferred to Hybond-N + membrane and hybridized with a randomly α-[^32^P]-dCTP-labelled dsDNA probe to detect *5.8S rRNA* and a strand-specific oligonucleotide probe labeled with α-[^32^P]-dATP to detect 7S RNA. The membrane was used to expose a PhosphorImager screen.

### *In organello* transcription assay

Freshly isolated mitochondria (1 mg) from mouse heart were washed twice in 0.5 ml of cold incubation buffer (see the *in organello* replication assay) supplemented with 1 mg/ml of fatty acid-free BSA and 1 mM ADP. Next, mitochondria were resuspended in 1 ml of prewarmed incubation buffer supplemented with 1 mg/ml of fatty acid-free BSA, 1 mM ADP and 60 μCi α-[^32^P]-UTP. Samples were further incubated 1.5 h at 37°C on a rotating wheel. Mitochondria were pelleted, resuspended again in prewarmed incubation buffer containing 1 mg/ml of fatty acid-free BSA, 1 mM ADP and non-radioactive UTP and incubated for an additional 10 min at 37°C. Finally, samples were washed three times in washing buffer (see the *in organello* replication assay). The mtRNAs were extracted using the TRIzol/chloroform extraction method. Samples were separated in 1% MOPS-formaldehyde agarose gel, transferred to Hybond-N+ membrane and the signal was visualized by autoradiography. To ensure equal input, aliquots of mitochondrial proteins, taken before mtRNA isolation, were separated by SDS-PAGE and transferred to a PVDF membrane which was hybridized with an antibody against the mitochondrial HSP60 protein.

### Transcriptome and small RNA-seq analyses

Total RNA was extracted from control and *Rnaseh1* knockout hearts from 24-week-old mice by using the Qiagen miRNeasy kit according to the kit instructions for transcriptome analysis. Mitochondrial RNA was extracted with the same kit from isolated heart mitochondria of 24-week-old mice for small RNA-seq analysis. For TruSeq, paired-end reads were aligned to the mouse genome (mm10) with HISAT2 (32) v2.10 using default parameters. Properly paired, primary alignments were retained and read pairs aligned to the mitochondrial genome were realigned with the –no-spliced-alignment parameter enabled to remove spurious mitochondrial splice junctions. Gene counts were produced with featureCounts (33) v1.6.3 (-C -p -B -P -s 2) using the Ensembl GRCm38 v94 annotation combined with an in-house mitochondrial annotation. Differential expression was analyzed with DESeq2 (34) v1.18.1 in R v3.5.1. For small RNA, single-end reads were aligned to the mouse genome (mm10) with bowtie2 (35) v2.3.4.1 using default parameters. Gene counts were produced with featureCounts v1.6.3 (-s 1) using the Ensembl GRCm38 v94 annotation combined with an in-house mitochondrial annotation. Differential expression was analyzed with DESeq2 v1.18.1 in R v3.5.1. Strand-specific coverage profiles were generated with samtools (36) and bedtools (37) genomecov and normalized to total reads mapped to mitochondria and the generated data was visualised with Circos (38).

### Western blot analyses

For western blot analysis, mitochondria from the mouse heart or total-cell lysates from skin-derived fibroblasts (10–25μg) were resuspended in 4x Lämmli buffer (4% SDS, 20% glycerol, 120 mM Tris, 0.02% Bromophenol Blue), separated by SDS-PAGE (4–12% Bis–Tris Protein Gel, Invitrogen) and transferred to PVDF membranes. Immunoblotting was done according to standard procedures. Primary antibodies used for immunoblotting are listed in the Supplementary Table S1.

### BN-PAGE and *In-gel* activity assays

Around 75 μg of isolated mitochondria were lysed in 50 μl of solubilization buffer [20 mM Tris–HCl (pH 7.4), 0.1 mM EDTA, 50 mM NaCl and 10% (v/v) glycerol containing 1% (w/v) digitonin] and mixed with loading dye [5% (w/v) Coomassie Brilliant Blue G-250, 150 mM Bis–Tris and 500 mM 6-aminocaproic acid (pH 7.0)]. Blue native–polyacrylamide gel electrophoresis samples were resolved on 3–13% gradient gels and further subjected to *in-gel* activity staining for complexes I, II, and IV. *In-gel* activity assays were performed as previously described (39).

### COX/SDH double-labeling enzyme histochemistry

Skeletal muscle sections were incubated in freshly prepared COX medium [100 μM cytochrome c, 4 mM diaminobenzidine tetrahydrochloride, catalase (20 μg/ml), and 0.2 M phosphate buffer (pH 7.0)]. Following incubation for 45 min at 37°C, slides were washed three times in PBS. Next, slides were incubated in freshly prepared SDH medium [130 mM sodium succinate, 200 μM phenazinemethosulphate, 1 mM sodium azide, 1.5 mM nitroblue tetrazolium, and 0.2 M phosphate buffer (pH 7.0)] for 30 min at 37°C. Finally, slides were washed three times with PBS, dehydrated (following concentration of ethanol: 70%, 75%, 95% and 2 × 99.5%) and mounted for bright-field microscopy.

### Protein purification

The coding sequence of human RNase H1 carrying a C-terminal 6 × His-tag without the predicted mitochondrial targeting sequence (residues 1–26) was cloned into a pBacPAK9 vector (Clontech). The mutant RNase H1 versions, Y29C and V142I were generated by site-directed mutagenesis (Agilent Technologies). Recombinant wild-type and mutant human RNase H1 proteins were expressed in Sf9 insect cells as previously described (40). The proteins were purified over HIS-Select Nickel Affinity Gel (Sigma-Aldrich) pre-equilibrated with 10 mM imidazole in buffer A (25 mM Tris–HCl pH 7.5, 400 mM NaCl, 10% glycerol, 10 mM β-mercaptoethanol and 1× protease inhibitors), followed by elution with 250 mM imidazole in buffer A. The proteins were subsequently purified over HiTrap Heparin (GE Healthcare) followed by HiTrap SP (GE Healthcare). The HiTrap columns were equilibrated in 0.2 M NaCl purification buffer (25 mM NaHPO_4_ pH 7.1, 10% glycerol, 1 mM DTT and 1× protease inhibitors), and the proteins were eluted in a 20-ml salt gradient (0.2–1.2 M NaCl in purification buffer). All other recombinant proteins were expressed and purified as described previously (41).

### Nuclease activity assay

Nuclease activity reactions were performed on an 80 nt DNA template annealed to a 3′ end labelled 52 nt chimeric oligonucleotide that contained 26 ribonucleotides followed by 26 deoxyribonucleotides (26RNA:26DNA). The reactions (20 μl) contained 1× RNase H buffer (50 mM Tris–HCl pH 8.0, 75 mM KCl, 3 mM MgCl_2_ and 10 mM DTT), 20 fmol substrate, and increasing concentrations of wild-type or mutant RNase H1 proteins (10–300 fmol). The reactions were incubated at 37°C for 60 min and then stopped by the addition of 5 μl stop buffer [10 mM Tris–HCl pH 8.0, 0.2 M NaCl, 1 mM EDTA, 660 μg/ml glycogen (Roche), and 100 μg/ml proteinase K (Ambion)] followed by incubation at 42°C for 45 min. The samples were recovered by ethanol precipitation in the presence of 0.5-volume ammonium acetate (7.5 M) and dissolved in 10 μl of TE buffer. The samples were then treated with 300 mM KCl or KOH for 2 h at 55°C and stopped with 2× stop buffer (formamide with 10 mM EDTA, 0.025% bromophenol blue and 0.025% xylene cyanol).

### *In vitro* transcription

All transcription reaction volumes were 25 μl and contained 25 mM Tris–HCl pH 8.0, 10 mM MgCl_2_, 64 mM NaCl, 100 μg/ml BSA, 10 mM DTT, 400 μM ATP, 150 μM GTP, 150 μM CTP 10 μM UTP, 0.027 μM α-[^32^P] UTP (3000 Ci/mmol), 4 nM of indicated plasmid template, 20 nM POLRMT, 200 nM TFAM, 60 nM TFB2M and 40 nM TEFM when indicated. The reactions were incubated at 32°C for 5 min and stopped by the addition of 200 μl stop buffer [10 mM Tris–HCl pH 8.0, 0.2 M NaCl, 1 mM EDTA, 100 μg/ml glycogen (Roche) and 100 μg/ml proteinase K (Ambion)] followed by incubation at 42°C for 45 min. The transcripts were recovered by ethanol precipitation and the pellets were dissolved in 20 μl gel loading buffer (98% formamide, 10 mM EDTA, 0.025% xylene cyanol FF and 0.025% bromophenol blue) and heated at 95°C for 3 min. The samples were analyzed on 4% denaturing polyacrylamide gels (1× TBE and 7 M urea) followed by exposure on photo film. Low Molecular Weight DNA Ladder (NEB) was used as a size marker.

### Replication initiation

All reaction volumes were 20 μl and contained 25 mM Tris–HCl pH 8.0, 10 mM MgCl_2_, 50 mM NaCl, 100 μg/ml BSA, 10 mM DTT, 400 μM ATP, 150 μM GTP, 150 μM CTP 150 μM UTP, 100 μM dATP, 100 μM dGTP, 100 μM dCTP (or ddCTP if indicated), 10 μM dTTP, 0.027 μM α-[^32^P]-dTTP (3000 Ci/mmol) and 8 nM of indicated template. All reactions (unless otherwise stated) contained 200 nM of TFAM, 60 nM of TFB2M, 20 nM of POLRMT, 20 nM of D274A POLγA exo- and 40 nM POLγB. Wild-type or mutant RNase H1 proteins were added in increasing concentrations as indicated (1, 2, 4 and 8 nM), and mtSSB was added at 40 nM unless otherwise stated. The reactions were incubated at 32°C for 30 min. Experiments with dCTP were stopped and evaluated as described for transcription reactions. Experiments with ddCTP were stopped by the addition of 5 μl stop buffer to a final concentration of 10 mM Tris-HCl pH 8.0, 0.2 M NaCl, 1 mM EDTA, 660 μg/ml glycogen (Roche) and 100 μg/ml proteinase K (Ambion) followed by incubation at 42°C for 45 min. The reactions were treated with KOH (300 mM) for 2 h at 55°C. The samples were recovered by ethanol precipitation in the presence of 0.5 volumes ammonium acetate (7.5 M), dissolved in 10 μl gel loading buffer (98% formamide, 10 mM EDTA, 0.025% xylene cyanol FF, and 0.025% bromophenol blue), and heated at 95°C for 3 min. The products were analyzed on 6% denaturing polyacrylamide gels (1× TBE and 7 M urea) for samples with dCTP or 12% denaturing polyacrylamide sequencing gels (1× TBE and 7 M urea) for samples with ddCTP. The gels were exposed on photo film.

### Oligonucleotides

For all *in vitro* studies, oligonucleotides were PAGE purified (Eurofins MWG Operon) with the exception of the primer extension oligonucleotide. Oligonucleotides were radiolabelled on the 3′-end. A 51 nt long 26RNA:25DNA oligonucleotide was radiolabelled to generate a 52 nt long 26RNA:26DNA oligonucleotide using terminal transferase (NEB) and α-[^32^P]-dCTP, followed by 3′ overhang removal using T4 DNA polymerase (NEB).

### Key resources and reagents

Key resources and reagents are listed in supplementary materials, Supplementary Table S1.

## RESULTS

### RNase H1 localizes to the mitochondrial matrix

**Figure 1.**
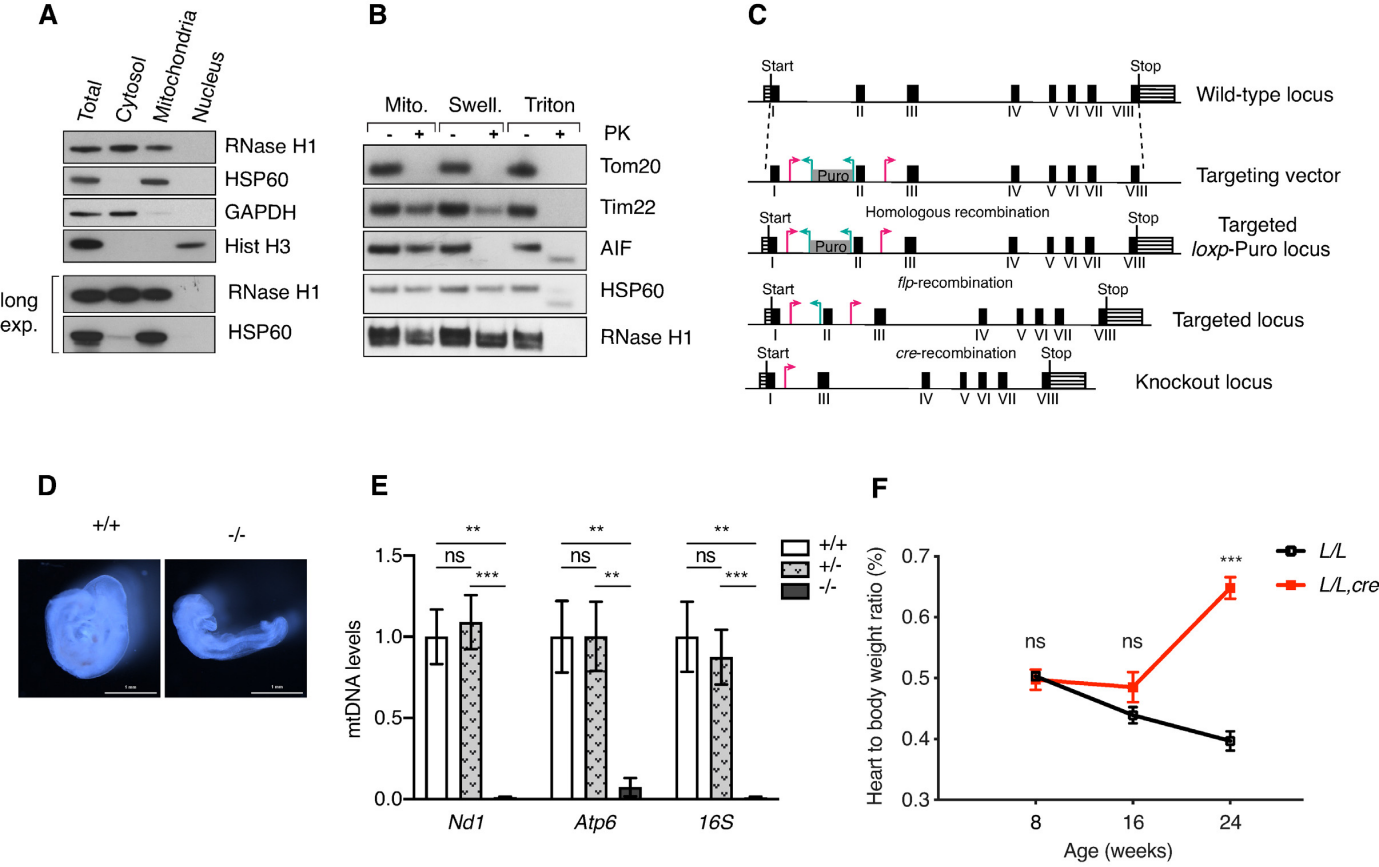
Disruption of *Rnaseh1* in the germline and heart. (**A**) Western blot analysis of subcellular fractionation of HEK293T cells. (**B**) Protease protection assay to assess submitochondrial localization of proteins. Mitochondria (Mito) were swollen in hypotonic buffer (Swell.) or lysed in a 1% triton X-100-supplemented buffer (Triton). Samples were treated (+) or left untreated (−) with proteinase K (PK). (**C**) Targeting strategy for disruption of the *Rnaseh1* gene. Red arrowhead: loxP sequence; green arrowhead: Frt sequence. (**D**) Morphology of *Rnaseh1^+/+^* and *Rnaseh1^–/–^* embryos at embryonic day 9.5. Scale bar 1 mm. (**E**) Steady-state levels of mtDNA in *Rnaseh1*^+/+^, *Rnaseh1*^+/–^ and *Rnaseh1^–/–^* embryos at E8.5 assessed with qPCR. 18S rDNA was used as a housekeeping gene control. Data are represented as mean ± SEM; Welch's *t*-test; *n* = 7–9 animals per group; *P < 0.05; **P < 0.01; ***P < 0.001. (**F**) Heart-to-body weight ratio of conditional knockout mice (*L/L, cre*) and controls (*L/L*). Data are represented as mean ± SEM; Welch's *t*-test; *n* = 5–11 animals of each genotype per age group; *P < 0.05; **P < 0.01; ***P < 0.001.

RNase H1 has been reported to be localized to both the nucleus and mitochondria in mice and humans (5,15). However, when we performed subcellular fractionation of human embryonic kidney cells (HEK293T), we found that RNase H1 was present in the mitochondrial and cytosolic fractions but it was not detected in the nuclear fraction (Figure 1A). To determine the submitochondrial localization of RNase H1, we performed protease protection assays and found that RNase H1 was not degraded by proteinase K added to mitochondria with an intact or disrupted outer membrane. The outer membrane protein Tom20 and the intermembrane space protein AIF were degraded in mitochondria and mitoplasts, respectively. Upon lysis of the inner membrane by detergent treatment, RNase H1 and the mitochondrial matrix protein HSP60 were degraded by proteinase K (Figure 1B). These findings show that RNase H1 is present in the mitochondrial matrix. A recent high-confidence mitochondrial proteome of HEK293 cells provides independent support for mitochondrial localization of RNase H1 (42). It is important to point out that our findings cannot exclude that a minor portion of RNase H1 also may be localized to the nucleus.

### RNase H1 is essential for embryo development and maintenance of adult differentiated tissues in mice

To study the *in vivo* role of RNase H1, we generated a conditional mouse knockout allele where exon II of the *Rnaseh1* gene was flanked with loxP sites (Figure 1C). Heterozygous knockout mice (*Rnaseh1^+/^^–^*) were obtained by crossing *Rnaseh1^+/loxP^* mice with *β-actin-cre* expressing mice. Intercrosses of *Rnaseh1^+/^^–^* mice produced no viable homozygous knockouts (genotyped offspring = 117; *Rnaseh1^+/+^*, *n* = 47; *Rnaseh1^+/^^–^*,*n* = 70; *Rnaseh1^–^^/^^–^*,*n* = 0). In line with a previous study (15), we observed that *Rnaseh1* was essential for embryonic development. Homozygous knockout embryos (*Rnaseh1^–^^/^^–^*) were small and poorly developed in comparison with wild-type (*Rnaseh1^+/+^*) embryos at E9.5 (Figure 1D). Furthermore, embryos analyzed at E8.5 had an almost complete depletion of mtDNA (Figure 1E). To study the *in vivo* role of RNase H1 in a differentiated tissue, we proceeded to generate mice with disruption of *Rnaseh1* in heart and skeletal muscle (Genotype: *Rnaseh1^loxP/^*^loxP^; +/*Ckmm-*cre; hereafter denoted *Rnaseh1* knockout mice). Reverse transcription PCR (RT-PCR) analyses showed that exon 2 was lacking in *Rnaseh1* transcripts in the knockout hearts (Supplementary Figure S1A). And the real-time quantitative PCR (qPCR) showed that *Rnaseh1* transcript levels were significantly reduced (Supplementary Figure S1B), consistent with degradation of the *Rnaseh1* transcripts by nonsense-mediated RNA decay in *Rnaseh1* knockout hearts. The *Rnaseh1* knockout mice had reduced lifespan with maximal longevity of ∼24 weeks (Supplementary Figure S1C). From 8 weeks onwards, *Rnaseh1* knockout animals developed a gradual increase in the heart to body weight ratio indicating progressive cardiomyopathy (Figure 1F). These results establish that RNase H1 is essential for both embryonic development and normal heart function in mice.

### mtDNA depletion and formation of linear deleted mtDNA in RNase H1 knockouts

**Figure 2.**
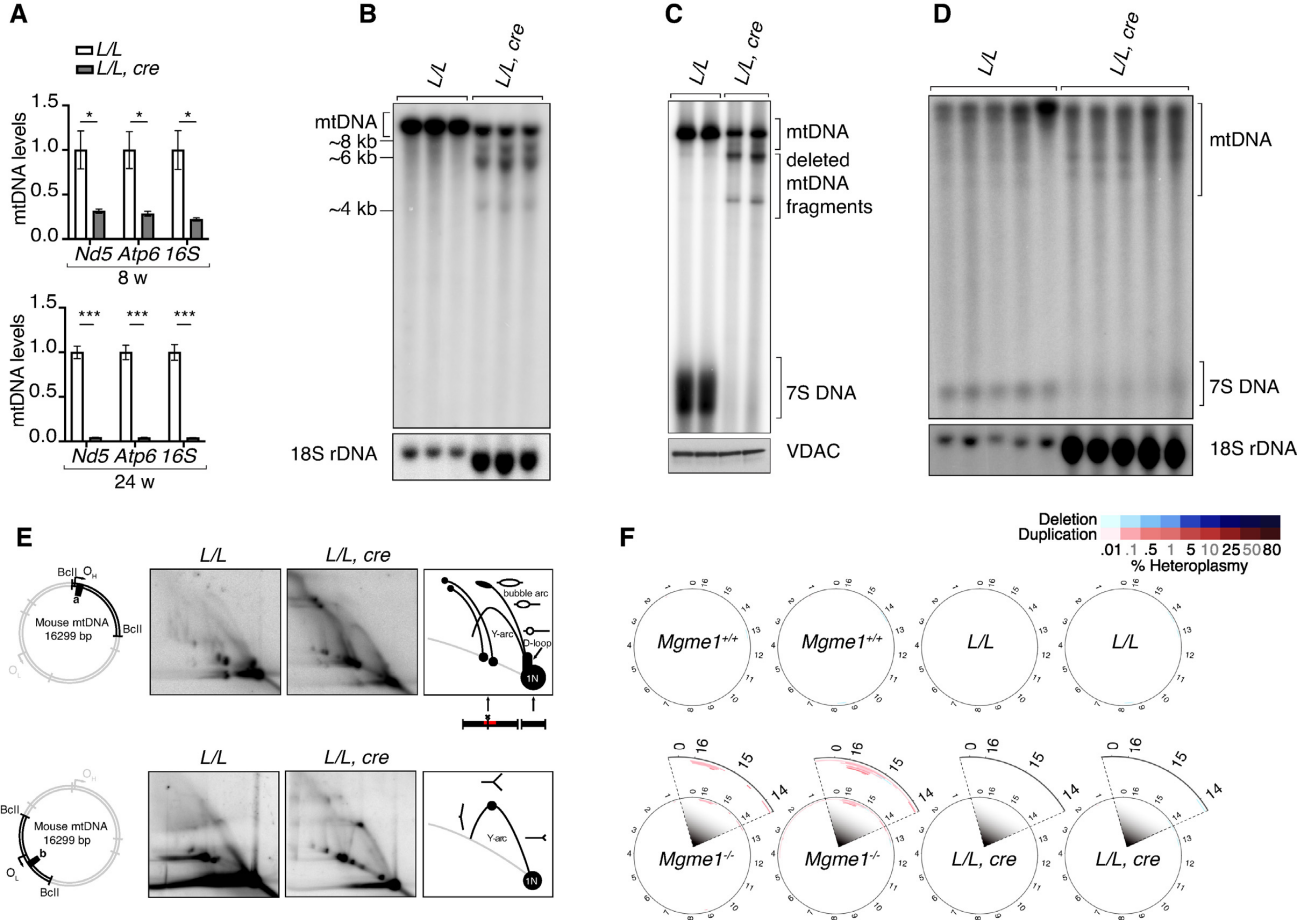
Loss of RNase H1 leads to mtDNA depletion and accumulation of deleted mtDNA fragments in mouse heart. (**A**) Relative mtDNA levels in 8- and 24-week-old control (*L/L)* and knockout (*L/L, cre)* mice as determined by qPCR on total heart DNA. 18S rDNA was used as a housekeeping gene control. Data are represented as mean ± SEM; Welch's *t*-test; *n* = 5 animals per group; *P < 0.05; **P < 0.01; ***P < 0.001. (**B**) Southern blot analysis of SacI-digested total DNA from heart of 16-week-old *L/L* and *L/L, cre* mice. Plasmid pAM1, containing whole mtDNA sequence was used as a probe. 18S rDNA was used as a loading control. (**C**) *De novo* DNA synthesis in heart mitochondria of 8-week-old *L/L* and *L/L, cre* mice. Radioactively labelled mtDNA was digested with SacI. The input was ensured by western blot analysis (VDAC) on isolated mitochondria after labeling before mtDNA extraction. (**D**) Steady-state levels of 7S DNA in 16-week-old *L/L* and *L/L, cre* mice by Southern blot analysis on total heart DNA. 18S rDNA was used as a loading control. For better comparison and visualization the loaded amount of total DNA from *L/L, cre* samples was 5 times more in (**B**) and 7 times more in (**D**) than the amount loaded from *LL* samples. (**E**) mtDNA replication pattern in the heart of 16-week-old *L/L* and *L/L, cre* mice by 2DNAGE followed by Southern blot. BclI-restriction sites and probes used are indicated to the left. The black bars denote probes used (*n* = 3 animals per group), O_H_ indicates origin of H-strand replication and O_L_ marks origin of L-strand replication. (**F**) mtDNA deletions (blue) and duplications (red) predicted from next generation sequencing data in *L/L* and *L/L, cre* hearts (*n* = 2 animals per group). Color intensity represents heteroplasmy level as indicated. Zoom-in region highlights duplication events predicted in *Mgme1^–/–^* brain samples. Mitochondrial genome coverage of each sample is labeled on the plots.

Analysis of mtDNA levels by qPCR in hearts lacking RNase H1 revealed a marked decrease at 8 weeks of age (∼22–31% of wild-type levels), progressing to a profound reduction at 24 weeks of age (∼4% of wild-type levels) (Figure 2A). The mtDNA depletion observed by qPCR was confirmed by Southern blot analyses (Figure 2B and Supplementary Figure S1D). Interestingly, linear deleted mtDNA fragments were present on Southern blots of RNase H1 knockout hearts (Figure 2B, Supplementary Figure S2A–F) and skeletal muscle (Supplementary Figure S1D). The linear deleted mtDNA fragments are of a similar size as the ones previously reported in patients and mice lacking the Mitochondrial Genome Maintenance Exonuclease 1 (MGME1), and in mtDNA mutator mice that express an exonuclease-deficient mitochondrial DNA polymerase (POLγ) (43–45). Loss of RNase H1 thus leads to mtDNA depletion and accumulation of high levels of linear deleted mtDNA fragments.

### Defective mtDNA replication in *Rnaseh1* knockout mice

The depletion of mtDNA and the aberrant replication product in *Rnaseh1* knockout mice prompted us to further investigate the regulation of mtDNA replication. Analysis by *in organello* replication assays showed that linear deleted mtDNA fragments were generated as by-products during ongoing mtDNA replication in heart mitochondria lacking RNase H1 (Figure 2C). To further investigate mtDNA replication, we assessed the steady-state levels of 7S DNA, the short nascent DNA molecule formed by premature replication termination at the end of the control region resulting in D-loop formation (46). Interestingly, we found a drastic reduction of 7S DNA levels in mouse hearts lacking RNase H1 even when Southern blots were overloaded to compensate for the mtDNA depletion (Figure 2D). Moreover, *in organello* mtDNA replication assays of heart mitochondria from *Rnaseh1* knockout mice showed no *de novo* synthesis of a distinct 7S DNA species (Figure 2C). Next, we performed 2D neutral-neutral agarose gel electrophoresis (2DNAGE) to analyze mtDNA replication products initiated from O_H_ and found increased levels of stalled mtDNA replication intermediates (Figure 2E), consistent with generalized inhibition of mtDNA replication progression. The 2DNAGE analysis probed with a restriction fragment containing O_H_ showed the presence of high molecular weight arcs resulting from blockage of the restriction sites, presumably due to unprocessed RNA primers still annealed to the DNA at the BclI restriction site immediately downstream of the light strand promotor (LSP) (Figure 2E). We also observed site-specific stalling near O_L_ in the absence of RNase H1 (Figure 2E). To develop a global overview of putative mtDNA alterations in the *Rnaseh1* knockout mice, we performed deep sequencing using the recently developed Mitochondrial Structural Alterations (MitoSAlt) high-throughput computational pipeline for analysis (30). We found no circular mtDNA molecules with deletions or mtDNA duplications in the *Rnaseh1* knockout hearts (Figure 2F), whereas simultaneous analysis of *Mgme1* knockout brains, used as a control, identified multiple, short duplications in the non-coding region (NCR), as previously described (30). Our results thus show that the marked mtDNA depletion in *Rnaseh1* knockout mice is a direct consequence of mtDNA replication defects.

### RNase H1 directs site-specific primer formation for initiation of mtDNA replication

**Figure 3.**
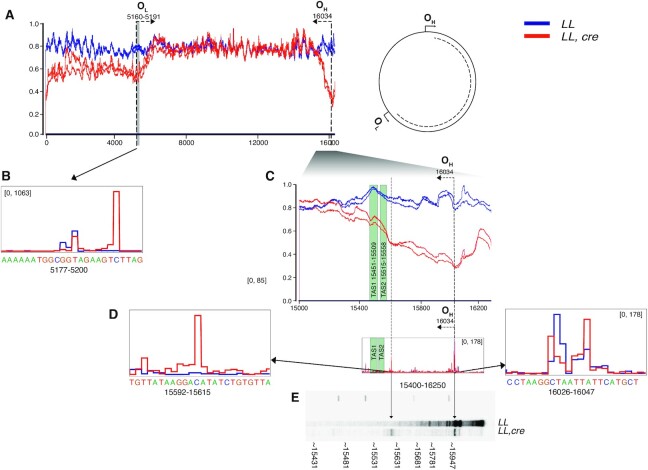
Altered replication initiation in absence of RNase H1. (**A**) To the left, sequence coverage of the heart mtDNA samples from control (*L/L)* (blue) and knockout (*L/L, cre)* (red) mice. x-axis shows the mitochondrial genome position and y-axis shows sequence coverage divided by maximum coverage for each sample. The approximate locations of the origins of L strand (O_L_) and H strand (O_H_) replication are indicated by grey box and dashed lines with arrows. *n* = 2 animals per group. To the right, schematic representation of circular mitochondrial genome with linear deletion highlighted as dotted line. (**B**) Position of 5′ DNA ends by HydEn sequencing of heart mtDNA from *L/L* (blue) and and *L/L, cre* (red) mice in proximity of O_L_. (**C**) Zoom-in image of sequence coverage (coordinate 15 000–16 299) shown in (**A**). Termination associated sequence (TAS) regions are indicated by green boxes. (**D**) Position of 5′ DNA ends by HydEn sequencing of heart mtDNA from *L/L* (blue) and and *L/L, cre* (red) mice in the control region. For HydEn sequencing data (**B–D**) sequence coverage is shown as an average of three samples derived from different mouse hearts. (**E**) Primer extension of heart mtDNA from *L/L* and and *L/L, cre* mice with primer corresponding to mtDNA positions 15 181–15 201.

We performed next-generation sequencing of *Rnaseh1* knockout heart DNA to map the ends of the linear deleted mtDNA molecules. The linear deletions were shown to contain sequences corresponding to the major arc region of mtDNA (Figure 3A), similar to the findings in MGME1 knockout mice (43). However, the regions close to O_H_ and O_L_ were comparatively underrepresented in the linear deleted mtDNA molecules from *Rnaseh1* knockout hearts (Figure 3A). This sharp decrease in sequence coverage observed at O_L_ (Figure 3A) is in line with the replication stalling we observed at O_L_ on 2DNAGE (Figure 2E). We proceeded to use hydrolytic DNA end sequencing (HydEn) (19,31) and found peaks indicating DNA ends located a few nucleotides downstream of O_L_ in the *Rnaseh1* knockout hearts (Figure 3B). Analysis of HydEn-seq data in the D-loop region revealed peaks at O_H_ in both wild-type and *Rnaseh1* knockout hearts (Figure 3D). However, additional peaks were found in the termination associated sequence (TAS) region in the *Rnaseh1* knockout hearts (Figure 3D), pointing to novel 5′ DNA ends. These peaks are in the region where a drop in mtDNA sequence coverage is observed (Figure 3A, C). As an independent means of detecting 5′ DNA ends of the nascent H-strand, we performed primer extension assays of DNA from wild-type and *Rnaseh1* knockout hearts. Primer extension with a primer complementary to the H-strand of the *CytB* gene (position 15181–15201) generated terminated products at different locations in the D-loop region in *Rnaseh1* knockout hearts that were absent in control mice (Figure 3E). These results are consistent with the presence of alternate sites for initiation of mtDNA replication in the D-loop region of *Rnaseh1* knockouts (Figure 3A, C–E). RNase H1 is thus essential for site-specific restriction of RNA replication primer formation to allow accurate initiation of DNA replication at both O_L_ and O_H_.

### Severe mitochondrial dysfunction in *Rnaseh1* knockout mice

**Figure 4.**
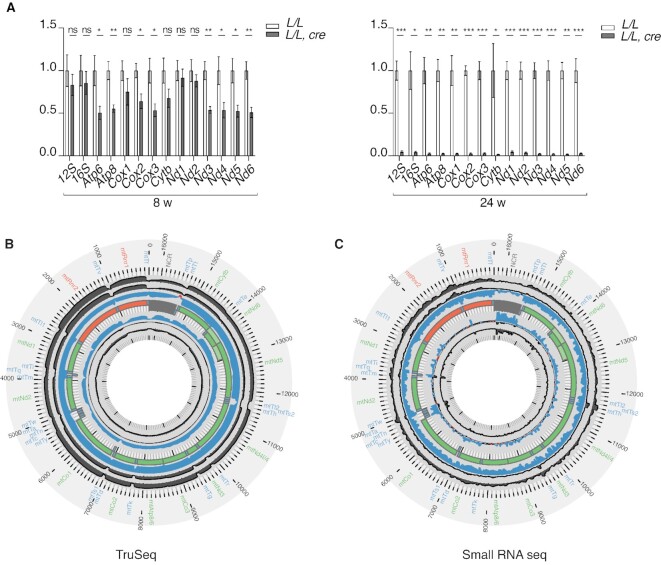
Decreased mitochondrial transcript levels in *Rnaseh1* knockout. (**A**) Steady-state levels of mtDNA-encoded transcripts in 8- and 24-week-old control (*L/L)* and knockout (*L/L, cre)* mice determined using RT-qPCR on total heart RNA. Actin B was used as a housekeeping gene control. Data are represented as mean ± SEM; Welch's *t*-test; *n* = 5 animals per group; *P < 0.05; **P < 0.01; ***P < 0.001. (**B**) TruSeq. Changes in the mitochondrial transcriptome from heart at 24 weeks of age as determined by RNA-Seq coverage on the heavy (outer track) and light (inner track) strands. Increases in red and decreases in blue (log_2_[RPM_KO_/RPM_WT_]). The central track is mtDNA; rRNAs (orange), mRNAs (green), tRNAs (blue) and the noncoding region NCR (grey) are indicated (*n* = 4 animals per group). (**C**) Small RNA seq. Changes in the mitochondrial transcriptome as determined by small RNA-Seq coverage from hearts at 24-week-old of age. Increases in red and decreases in blue (log_2_[RPM_KO_/RPM_WT_]). The central track is mtDNA; rRNAs (orange), mRNAs (green), tRNAs (blue) and the noncoding region NCR (grey) are indicated (*n* = 4 animals per group).

**Figure 5.**
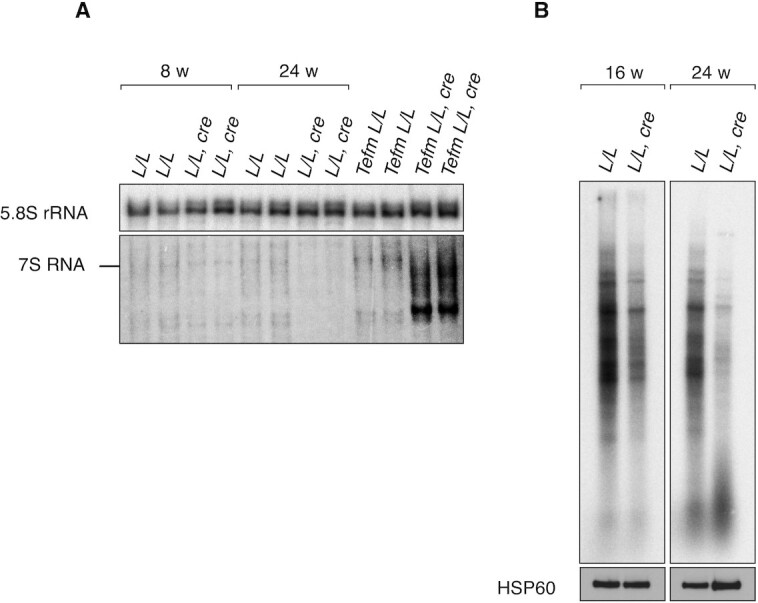
Reduced *de novo* transcription in *Rnaseh1* knockout hearts. (**A**) Northern blot analysis of 7S RNA from total heart RNA of 8- and 24-week-old control (*L/L)* and knockout (*L/L, cre)* mice and *Tefm*^*L/L*^ (control) and *Tefm^L/L, cre^* (knockout) mice. 5.8S rRNA was used as a loading control. (**B**) *De novo* synthesized mtDNA-encoded transcripts in heart mitochondria from 16- and 24-week-old *L/L* and *L/L, cre* mice. The input was ensured by western blot analysis (HSP60) on isolated mitochondria after labeling before RNA extraction.

Given reports that RNase H1 is important for removal of RNA:DNA hybrids formed during transcription of mtDNA (20,22), we monitored the effects of *Rnaseh1* knockout on mitochondrial gene expression. Consistent with the progressive mtDNA depletion (Figure 2A), the steady-state levels of mitochondrial transcripts in hearts of *Rnaseh1* knockout mice were slightly lower at 8 weeks and drastically lower at 24 weeks of age (Figure 4A). TruSeq and small RNA sequencing showed a general decrease in both long and short mtDNA-encoded transcripts in 24-week-old *Rnaseh1* knockout mice (Figure 4B, C). No processing defects of mitochondrial transcripts were found (Figure 4B). Northern blot analyses showed a similar age-dependent reduction in the steady-state levels of 7S RNA in the knockout hearts (Figure 5A), correlating with the decrease of mtDNA levels (Figure 2A). The mitochondrial transcription elongation factor (TEFM) is required for the transition from transcription initiation to elongation, and in its absence a range of abnormally short 7S RNAs accumulate (47) (Figure 5A). In contrast, loss of RNase H1 does not appear to affect transcription elongation as only full length 7S RNAs were found (Figure 5A). Consistent with these results, there is a gradual decline in the abundance of promoter-distal transcripts in heart- and skeletal-muscle-specific *Tefm* knockout mice (47), but this pattern was not seen in *Rnaseh1* knockouts (Figure 4B, C) further arguing against a transcription elongation defect in the absence of RNase H1. *In organello* transcription assays also showed significantly decreased *de novo* transcription in isolated heart mitochondria of *Rnaseh1* knockout mice at the age of 16 and 24 weeks (Figure 5B). Loss of RNase H1 thus leads to decreased levels of mitochondrial transcripts, likely secondary to the decrease of mtDNA templates, without evidence for defective transcription elongation or impaired transcript processing.

Consistent with the depletion of mtDNA and the global decrease in the levels of mtDNA-encoded transcripts, the steady-state levels of OXPHOS subunits were profoundly decreased in 24-week-old *Rnaseh1* knockout mice (Supplementary Figure S3A). Histochemical analysis of the combined cytochrome *c* oxidase/succinate dehydrogenase (COX/SDH) enzyme activity in skeletal muscle showed profound COX deficiency in *Rnaseh1* knockout mice (Supplementary Figure S3B). On blue native polyacrylamide gels (BN-PAGE), there was a profound decrease in complex I and IV (COX) activity in *Rnaseh1* knockout hearts at the age of 24 weeks (Supplementary Figure S3C). As expected, the levels of mitochondrial transcription factor A (TFAM), which normally follow the levels of mtDNA (48,49), and the levels of the leucine-rich pentatricopeptide repeat protein (LRPPRC), which typically follows the mitochondrial mRNA levels (50), were both decreased in hearts of 24 weeks old *Rnaseh1* knockout mice (Supplementary Figure S3A). In contrast, the levels of mitochondrial RNA polymerase (POLRMT) were increased (Supplementary Figure S3A), indicating that loss of RNase H1 leads to compensatory transcription increase, as seen in other mouse mutants affecting mtDNA expression (47,51,52).

In summary, our results demonstrate that loss of RNase H1 affects mtDNA replication in mice, which, in turn, leads to a severe decrease of mitochondrial transcript levels and profoundly impaired OXPHOS capacity.

### A novel pathogenic mutation affecting the hybrid-binding domain of RNase H1

**Figure 6.**
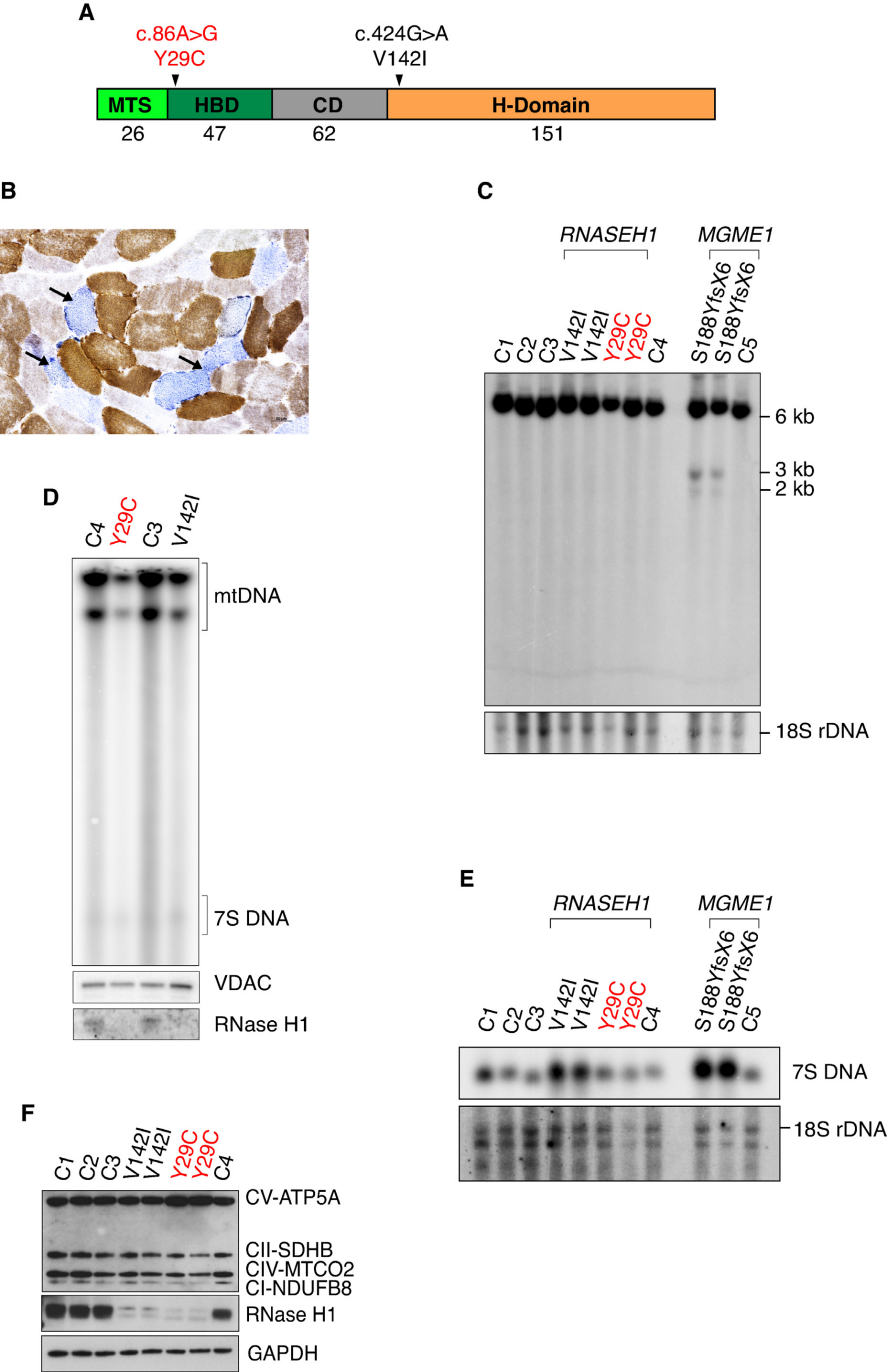
RNase H1 disease mutants. (**A**) Domain organization of human RNase H1 with the Y29C and V142I mutations indicated. The mitochondrial targeting signal (MTS), hybrid-binding domain (HBD), connection domain (CD), catalytic (H) domain are indicated. (**B**) COX/SDH staining of skeletal muscle biopsy from Y29C patient. Numerous COX-negative fibers are indicated by black arrows. Magnification 20x, scale bar 50 μm. (**C**) Steady-state levels of mtDNA in patients by Southern blot analysis on total fibroblast DNA. DNA was digested with EcoRI and BamHI. CytB was used as a probe and 18S rDNA was used as a loading control. (**D**) *De novo* DNA synthesis in mitochondria of skin fibroblast derived from Y29C and V142I patients and controls. The input was ensured by western blot analysis (VDAC) on isolated mitochondria after labeling before mtDNA extraction. (**E**) 7S DNA levels in patients by Southern blot analysis on total fibroblast DNA. 18S rDNA was used as a loading control. (**F**) Steady-state protein levels in patient fibroblasts by western blot analysis on total cell extracts.

An important insight into the *in vivo* function and mechanism of action of RNase H1 comes from studies in patients with pathogenic mutations in *RNASEH1* reducing enzyme activity. These patients often have mtDNA depletion similar to the findings in *Rnaseh1* knockout mice (15) (Figure 2A). However, unlike *Rnaseh1* knockout mice, patients with *RNASEH1* mutations do not accumulate linear deleted mtDNA molecules but instead have high amounts of circular deleted mtDNA molecules in skeletal muscle (23–26). This difference may be explained by the complete absence of RNase H1 in the knockout mice, whereas patients express an RNase H1 protein with impaired function. In contrast, most patients described with *MGME1* variants carry loss-of-function mutations, potentially explaining the greater similarity in mtDNA phenotypes between patients and knockout mice (43,53). Patients with *RNASEH1* mutations are typically homozygous or compound heterozygous for mutations in the catalytic (H) domain or compound heterozygous for mutations in the H-domain and the connection domain (CD) (Figure 6A). In a recently diagnosed pair of siblings, sister and brother, we found a homozygous c.86A > G mutation (Y29C) in the region of the *RNASEH1* gene encoding the hybrid-binding domain (HBD) of RNase H1 (Figure 6A). No mutations have previously been described in this domain and both patients developed very similar phenotypes with parkinsonism and cognitive deterioration, which have not previously been reported in patients with pathogenic *RNASEH1* mutations. Also, classical mitochondrial phenotypes were observed in both siblings, i.e. ptosis, PEO, peripheral neuropathy, sensorineural deafness, and mitochondrial myopathy with abundant COX-negative fibers (Figure 6B), multiple mtDNA deletions (Supplementary Figure S4A) but normal mtDNA copy number (Supplementary Figure S4B). Southern blot analyses in fibroblasts from the Y29C patient and another patient with a previously described V142I mutation of RNase H1 also showed normal mtDNA content (Figure 6C) (23). However, *in organello* replication assays showed decreased *de novo* mtDNA synthesis in mitochondria of both Y29C and V142I patient-derived fibroblasts (Figure 6D), demonstrating that malfunction of either the HBD or H-domain of RNase H1 impairs mtDNA replication. As previously reported for patient fibroblasts carrying the V142I amino acid substitution of RNase H1 (23), no linear deleted mtDNA was seen in fibroblasts derived from the Y29C patient (Figure 6C). As a control sample, we included fibroblasts from a patient affected by early-onset PEO and mitochondrial myopathy with sparse COX-negative fibers (Supplementary Figure S4C) caused by a novel loss-of-function mutation (S188YfsX6) in *MGME1*. This patient had linear deleted mtDNA molecules in fibroblasts (Figure 6C) and skeletal muscle (Supplementary Figure S4A), similar to the findings in other patients lacking MGME1 (45). Interestingly, the steady-state levels of 7S DNA were increased in fibroblasts from the *RNASEH1* patient with the V142I mutation, as previously described (23), and remained normal in the patient with the Y29C mutation of RNase H1 (Figure 6E). A normal amount of 7S DNA was also observed in the skeletal muscle biopsy specimen (Supplementary Figure S4D). The patient with the S188YfsX6 mutation of MGME1 had increased levels of 7S DNA in both fibroblasts and skeletal muscle (Figure 6E and Supplementary Figure S4D) consistent with findings in other patients with *MGME1* mutations (53). In contrast to the findings in patients, *Rnaseh1* knockout mice showed a complete lack of 7S DNA (Figure 2C, D). The steady-state levels of RNase H1 protein were significantly reduced in fibroblasts from patients with the two different *RNASEH1* mutations (Figure 6F), showing that both the Y29C and V142I amino acid substitutions of RNase H1 impair not only protein function but also protein turnover.

### Tightly regulated RNase H1 activity is critical for replication initiation *in vitro*

**Figure 7.**
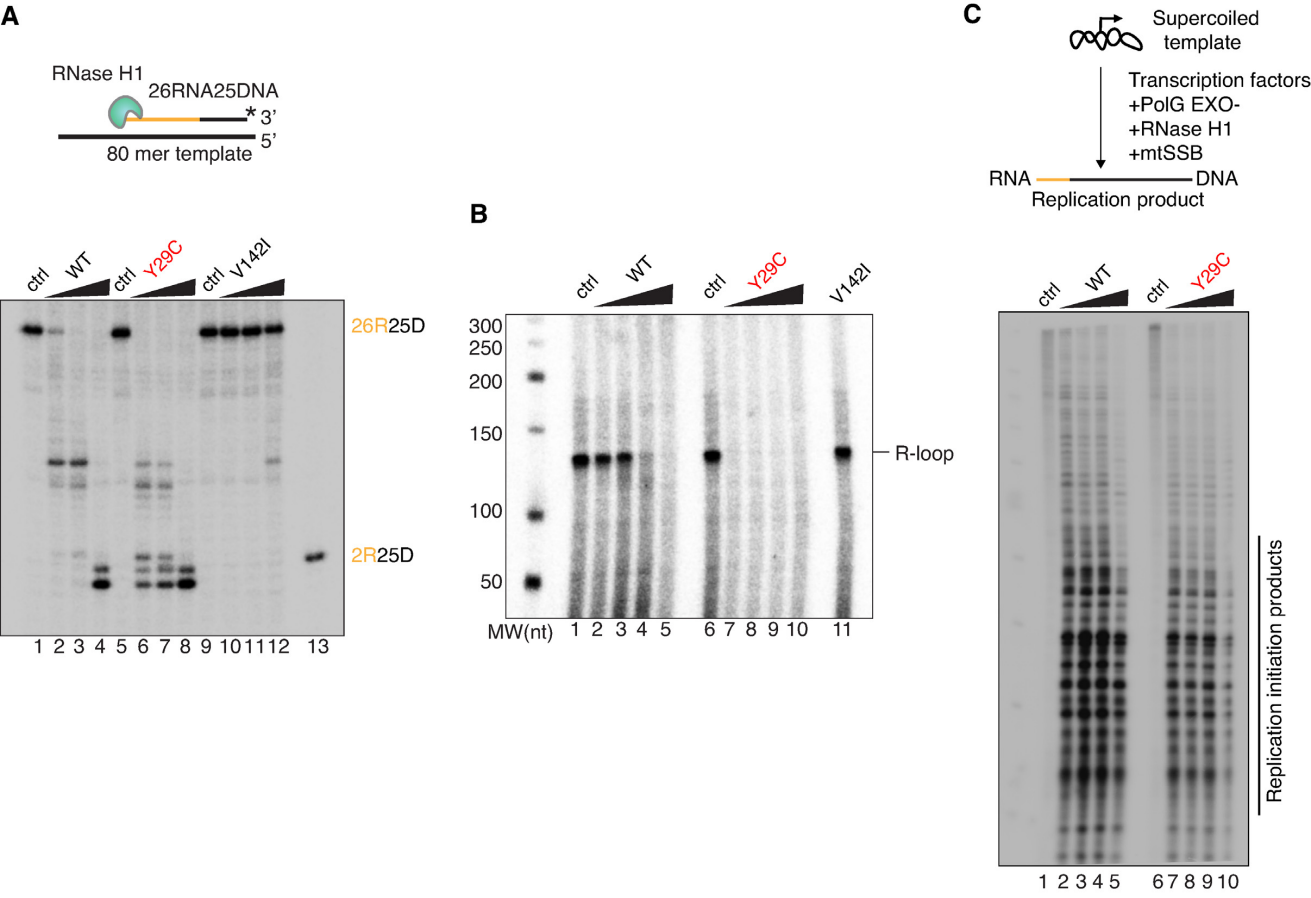
HBD mutant displays enhanced RNase H1 activity but supports reduced DNA replication initiation *in vitro*. (**A**) RNA–DNA hybrid processing by RNase H1 mutant enzymes. WT, Y29C and V142I enzymes were added at 1, 5 and 25 nM concentrations. Lanes 1, 5 and 9 are no RNase H1 controls. (**B**) Effects of RNase H1 mutant enzymes on *in vitro* transcription and R-loop processing. WT and Y29C enzymes were added at 0.5, 2, 8, 32 nM concentrations and V142I at 32 nM. Lanes 1 and 6 are no RNase H1 controls. (**C**) Replication initiation assay in absence (lanes 1 and 6) or increasing amounts of either WT or Y29C enzyme. Concentration curve 0.5, 2, 8, 32 nM.

Based on the different disease phenotypes, we compared the activity of the RNase H1 variants *in vitro*. First, we characterized the RNase H1, RNase H1:Y29C and RNase H1:V142I variants on a defined RNA:DNA hybrid template. RNase H1:Y29C was more active than the wild-type enzyme (Figure 7A), whereas RNase H1:V142I had a significantly reduced cleavage activity, as previously described (19). We have previously demonstrated that transcripts from LSP need to be processed by RNase H1 to promote site-specific initiation of mtDNA replication at O_H_ *in vitro* and we therefore investigated whether the mutant versions of RNase H1 can process R-loops formed by the transcription machinery. Surprisingly, we found that the activity of RNase H1:Y29C was increased relative to the wild-type enzyme, whereas RNase H1:V142I had significantly reduced cleavage activity (Figure 7A, B). However, when we employed an *in vitro* replication assay, fewer initiation products were formed by RNase H1:Y29C (Figure 7C) despite its increased processing activity on RNA:DNA templates (Figure 7A, B). Our findings thus show that the catalytic activity of RNase H1 does not necessarily correlate with its ability to form replication primers, and that the HBD and H-domain must act in a coordinated way to generate the RNA primers necessary for initiation of mtDNA replication.

## DISCUSSION

**Figure 8.**
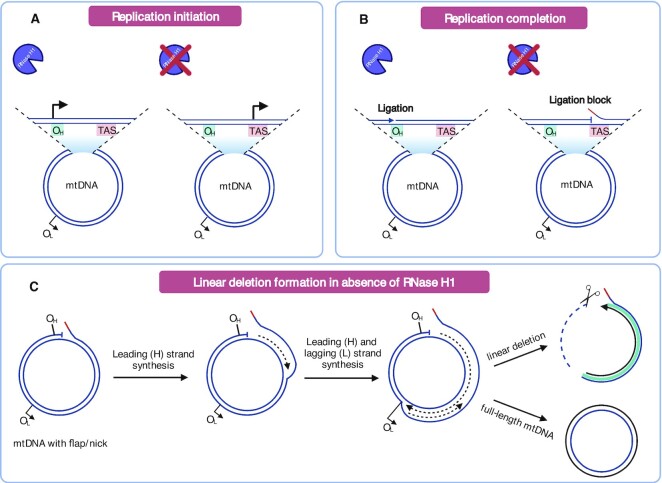
RNase H1 is essential for initiation and completion of mtDNA replication in mammalian mitochondria. (**A**) RNase H1 function is crucial to limit initiation of mtDNA replication to the replication origins, O_H_ and O_L_. In the absence of RNase H1, initiation of leading (H) strand synthesis at O_H_ does not occur and replication is instead initiated from a non-canonical site in the termination associated sequence (TAS) region. Likewise, loss of RNase H1 leads to lagging (L) strand synthesis from non-canonical sites downstream of O_L_ (not depicted). (**B**) RNase H1 is also required for replication completion of both the H and L strands (L strand replication completion is not shown). Loss of RNase H1 prevents digestion of the RNA primer residues attached to the 5′end of the nascent DNA strand. After completion of mtDNA replication the RNA will remain attached to the 5′ end of the DNA and will prevent DNA ligation. (**C**) Formation of linear mtDNA molecules with deletions in *Rnaseh1* knockout mice. Impaired ligation of the nascent H strand generates mtDNA molecules with a nick. In the next replication round, the nicked H strand becomes the template for L strand synthesis and replication of the L strand will therefore have an increased risk for premature termination near the TAS region. As a result, the ssDNA fragment spanning the minor mtDNA arc will be degraded leaving the linear, dsDNA fragment covering the major mtDNA arc.

Mammalian RNase H1 has an essential role in mtDNA replication (15), but it has been unclear at which exact step it acts *in vivo*. Here, we demonstrate that RNase H1 function is crucial for site-specific restriction of replication primer formation to limit initiation of mtDNA synthesis to the replication origins (Figure 8A). In addition, RNase H1 has a role during completion of mtDNA replication as demonstrated by the impaired replication fork progression and the formation of linear deleted mtDNA fragments in the absence of RNase H1 (Figure 8B, C).

According to the widely accepted strand-displacement model (SDM) of mtDNA replication (54), replication of the leading (H) strand commences at O_H_ and proceeds unidirectionally until approximately two thirds of the H-strand has been synthesized. At this point, the region surrounding O_L_ becomes single stranded and forms a stem loop structure where lagging (L) strand synthesis initiates. Replication initiation at O_H_ depends on transcription from LSP that results in an R-loop that is processed by RNase H1 to form RNA primers (19). The 3′ RNA ends that are formed after processing by RNase H1 are used by POLγ to initiate DNA synthesis (19). In the absence of RNase H1, R-loop structures formed by transcription from LSP remain unprocessed and origin-specific replication initiation does not occur at O_H_. Instead, we report here that mtDNA replication is initiated at specific non-canonical sites downstream of O_H_ and O_L_ in the absence of RNase H1 (Figure 8A). In agreement with our data, unspecific replication initiation from multiple sites unrelated to O_H_ was observed in fibroblasts from a patient with a homozygous disease-causing mutation in RNase H1 (19) and another replication initiation site, referred to as ori-b was reported in study in MEFs lacking RNase H1 (16). Moreover, the requirement for RNase H1 in the replication primer maturation process is conserved in evolution as it also has been demonstrated at the ColE1 replication origin in bacteria (55).

We have previously shown that mtSSB plays a major role in controlling the replication/transcription switch at O_H_ by promoting the formation and stabilization of the R-loop transcribed from LSP (52). In the absence of mtSSB, no R-loop is formed despite a strong increase in transcription from LSP, and mtDNA cannot be maintained at normal levels (52). These findings argue that mtSSB, in addition to its role in coating single-stranded mtDNA during replication, also provides a switch between transcription for primer formation and gene expression. In contrast, transcripts from LSP are terminated very close to the promoter in the absence of TEFM and both replication primer formation and transcription for gene expression are drastically impaired (47). Our data provide additional information on the mechanism for replication primer formation as we show that RNase H1 is necessary for generating the 3′ RNA-ends of the replication primers *in vivo*. Moreover, RNA-seq shows that RNase H1 is not important for removing R-loops, that potentially would block transcription elongation, or for ribosomal RNA processing, at variance with other reports (20–22). Given the potent strand displacement activity of POLRMT (56), we propose that this conserved structural feature may explain why POLRMT alone can be responsible for negotiating at least some R-loops formed as by-products of mitochondrial transcription.

In addition to the well documented role for RNase H1 in replication initiation, it is also likely important for mtDNA replication completion. When mtDNA replication has progressed around the circle, POLγ will encounter and displace the 5′ end where replication started to create a ∼100 nucleotides long DNA flap (57,58). Once replication is initiated, RNase H1 will cleave the remaining RNA primer hybridized to the template DNA. However, in the absence of RNase H1, the RNA primer will remain attached to the DNA flap displaced by POLγ (16). Normally, the DNA flap is processed by MGME1, but this processing may be impaired if the RNA primer remains attached to the 5′ end. It is important to recognize that impaired function of enzymes involved in determining the length and structure (POLγ, RNase H1) or degradation (MGME1) of the DNA flap all cause the formation of linear deleted mtDNA molecules extending between the two origins of mtDNA replication (43–45,59). Collectively, published studies and the results we present here strongly argue for the model where the linear deleted mtDNA molecules are formed during mtDNA replication as the result of persistent DNA flaps (60) or RNA residues attached to the 5′ end of the flaps that prevent ligation (Figure 8B). Ligation is likely impaired at both origins because of persisting DNA flaps, which means that a fraction of all mtDNA replication events cannot be completed causing the formation of linear deletions (Figure 8C). According to this model, the linear deleted mtDNA molecules are constantly formed but cannot undergo replication as they lack both origins of mtDNA replication.

Eukaryotic RNase H1 has an HBD, a CD and an H-domain (61) (Figure 6A). The HBD binds double-stranded RNA and RNA:DNA-hybrids (62–66) to ensure enzyme processivity. Deletion of the HBD generates an enzyme that has a more distributive cleavage activity, similar to the activity of the *E. coli* RNase H1 enzyme (65,66) that lacks the HBD. We show that a functioning HBD and H-domain are required for mammalian RNase H1 activity *in vivo*. Skin-derived fibroblasts from mitochondrial disease patients carrying mutations in the HBD or H-domain have defects in *de novo* mtDNA replication. Interestingly, mutations in the HBD and H-domain have opposite effects on the RNase H1 cleavage activity *in vitro*, causing significantly increased or drastically decreased enzyme activity, respectively. These results show that the RNase H1 activity must be tightly regulated *in vivo*. The HBD of RNase H1 may therefore not only have a role in controlling enzyme processivity, as previously proposed (65,66), but may also be necessary to position the enzyme for site-specific primer processing for mtDNA replication initiation.

In conclusion, we demonstrate that a carefully balanced RNase H1 activity is essential for regulating two crucial steps of mtDNA replication *in vivo*, i.e. replication initiation and replication completion. Lack of RNase H1 activity leads to mtDNA replication defects and the formation of linear deleted mtDNA fragments but does not directly affect transcription or posttranscriptional RNA processing in mammalian mitochondria.

## DATA AVAILABILITY

The mtDNA sequencing data, HydEn sequencing data and RNA sequencing data have been deposited in Gene Expression Omnibus under accession number GSE199771, GSE199198 and GSE199147, respectively.

## Supplementary Material

gkac661_Supplemental_File
