# Supplementary material for: Mammalian RNase H1 directs RNA primer formation for mtDNA replication initiation and is also necessary for mtDNA replication completion
Source: Nucleic Acids Res. 2022 Aug 10;50(15):8749–66. doi: 10.1093/nar/gkac661 (PMC9410905; doi:10.1093/nar/gkac661)
Supplement: gkac661_Supplemental_File [file gkac661_supplemental_file.pdf]

Supplementary Materials for

Mammalian RNase H1 directs RNA primer formation for mtDNA replication initiation and is also necessary for mtDNA replication completion

Jelena Misic, Dusanka Milenkovic, Ali Al-Behadili, Xie Xie, Min Jiang, Shan Jiang, Roberta Filograna, Camilla Koolmeister, Stefan J. Siira, Louise Jenninger, Aleksandra Filipovska, Anders R. Clausen, Leonardo Caporali, Maria Lucia Valentino, Chiara La Morgia, Valerio Carelli, Thomas J. Nicholls, Anna Wredenberg, Maria Falkenberg, Nils-Göran Larsson*

*Corresponding author. Email: nils-goran.larsson@ki.se

This PDF file includes:

Fig. S1. Tissue-specific *Rnaseh1* knockout mice.
Fig. S2. Linear deleted mtDNA in *Rnaseh1* knockout hearts.
Fig. S3. OXPHOS dysfunction in *Rnaseh1* knockout mice.
Fig. S4. mtDNA assessment in patient skeletal-muscle biopsy specimens.
Table S1. Key reagents and resources used in this study.

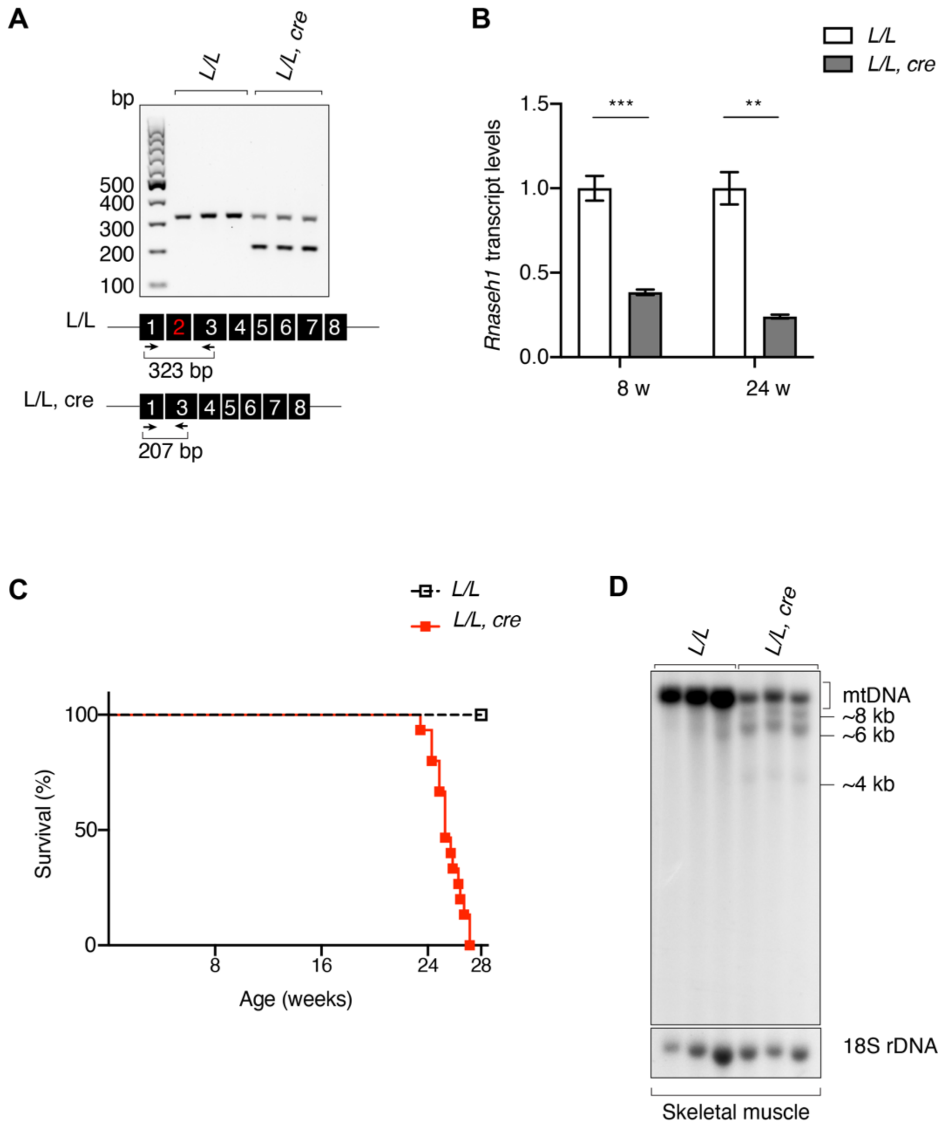

Fig. S1. Tissue-specific *Rnaseh1* knockout mice. (**A**), Schematic representation of *Rnaseh1* cDNA. RT-PCR analysis of *Rnaseh1* transcripts in heart of control (*L/L)* and knockout (*L/L, cre)* mice. (**B**), *Rnaseh1* transcript levels in heart of 8- and 24-week old *L/L* and *L/L, cre* mice by RT-qPCR. Actin B was used as a housekeeping gene control. Data are represented as mean ± SEM; Welch's t-test; n=5 animals per group; $*p < 0.05$; $**p < 0.01$; $***p < 0.001$. (**C**), Survival curve of *L/L* (n=15) and *L/L, cre* mice (n=15). Log-rank (Mantel-Cox) test was performed; p<0,0001. (**D**), Southern blot analysis of SacI-digested total DNA from skeletal muscle of 16-week-old *L/L* and *L/L, cre* mice. Plasmid pAM1, containing whole mtDNA sequence was used as a probe. 18S rDNA was used as a loading control.

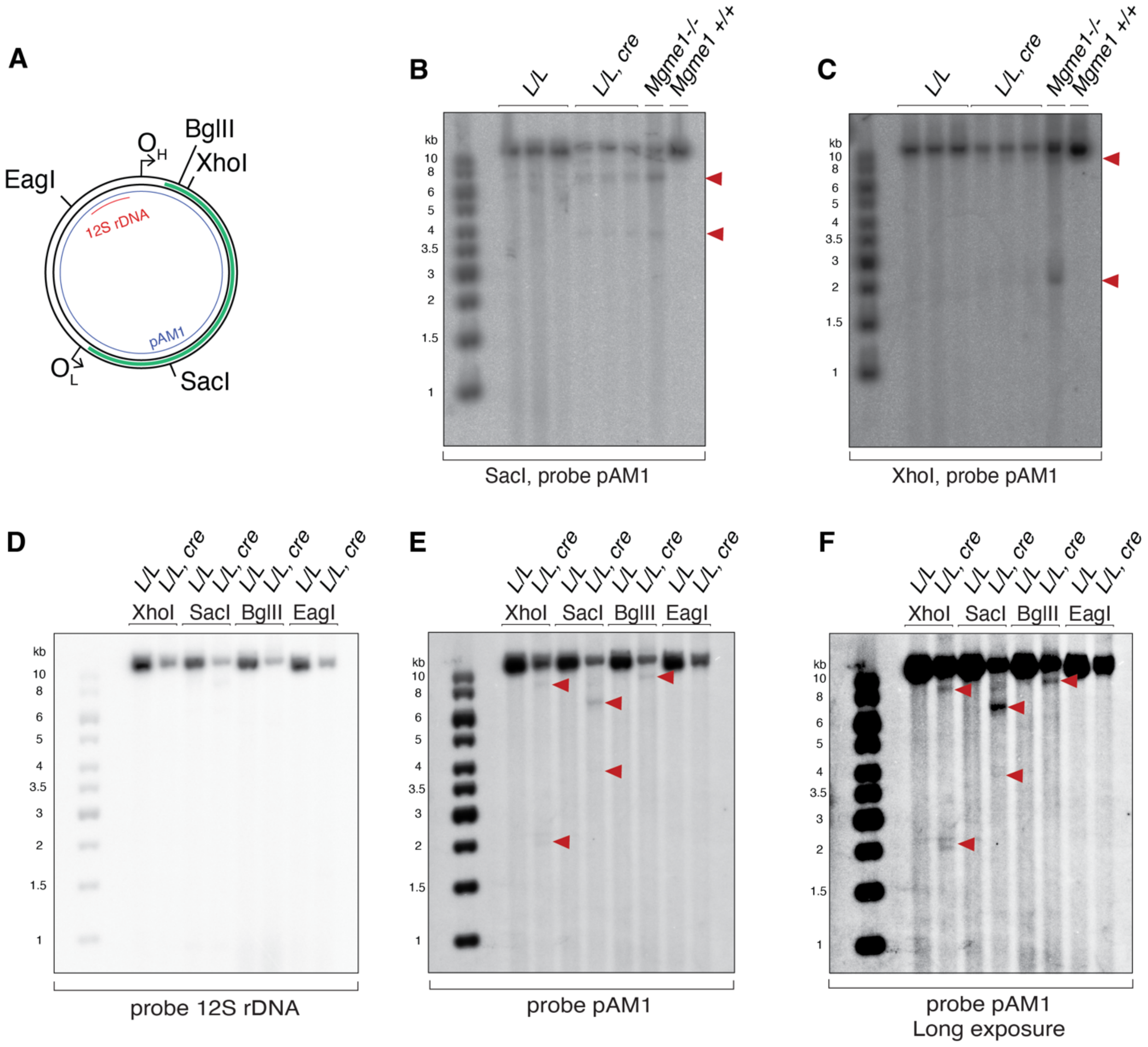

Fig. S2. Linear deleted mtDNA in *Rnaseh1* knockout hearts. (**A**), Schematic representation of mouse mtDNA with SacI, XhoI, BglII and EagI restriction sites. O_H indicates the heavy-strand origin of replication and O_L stands for the light-strand origin of replication. Probes used for following Southern blot analyses are represented in blue (pAM1) and in red (12S rDNA). Linear deleted mtDNA molecule spans the region highlighted in green. Southern blot analyses of heart mtDNA from control (*L/L*) and knockout (*L/L, cre)* 8-week-old mice and *Mgme1* $^{-/-}$ (knockout) and *Mgme1* $^{+/+}$(control) mice after digestion with SacI (**B**) or XhoI (**C**). Red arrows represent the DNA migration pattern of linear deleted mtDNA fragments in *Rnaseh1* and *Mgme1* knockout hearts. Southern blot analyses of *L/L* and *L/L, cre* heart mtDNA from 8-week-old mice. Samples were digested with one of the following enzymes: XhoI, SacI, BglII or EagI. Membrane was first hybridized with 12S rDNA probe (**D**), stripped and hybridized with pAM1 probe (**E** and **F**). Red arrows represent the DNA migration pattern of linear deleted mtDNA in *Rnaseh1* knockout hearts.

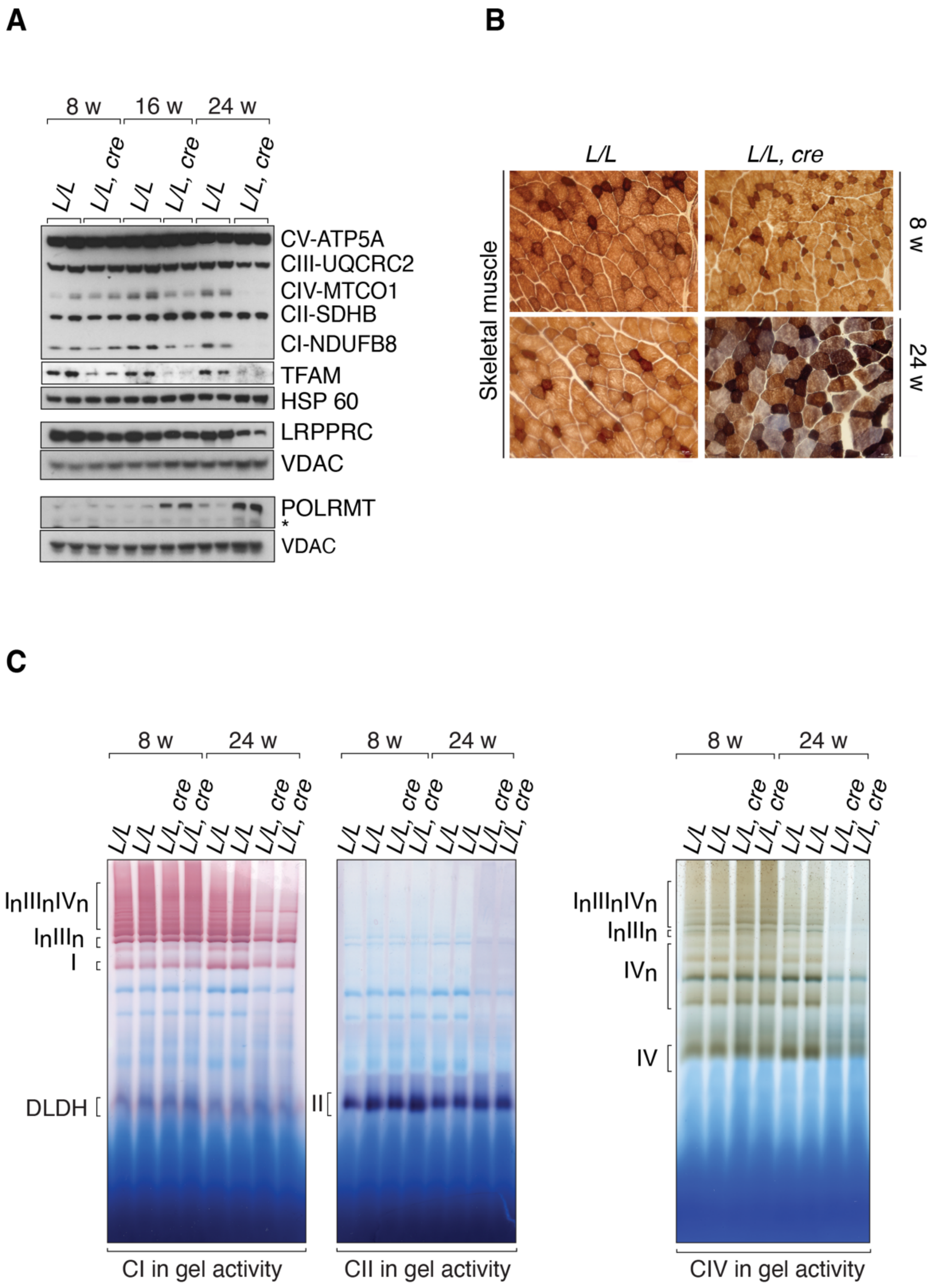

Fig. S3. OXPHOS dysfunction in *Rnaseh1* knockout mice. (**A**), Steady-state protein levels in mitochondria isolated from heart of 8-, 16- and 24-week-old control (*L/L)* and knockout (*L/L, cre)* mice by western blot analysis. (**B**), COX/SDH staining of skeletal muscle tissue from 8- and 24-

week-old *L/L* and *L/L, cre* mice. Scale bar 50 µm. (**C**), In gel activity of complex I, II and IV in mitochondria isolated from heart of 8- and 24-week-old *L/L* and *L/L, cre* mice.

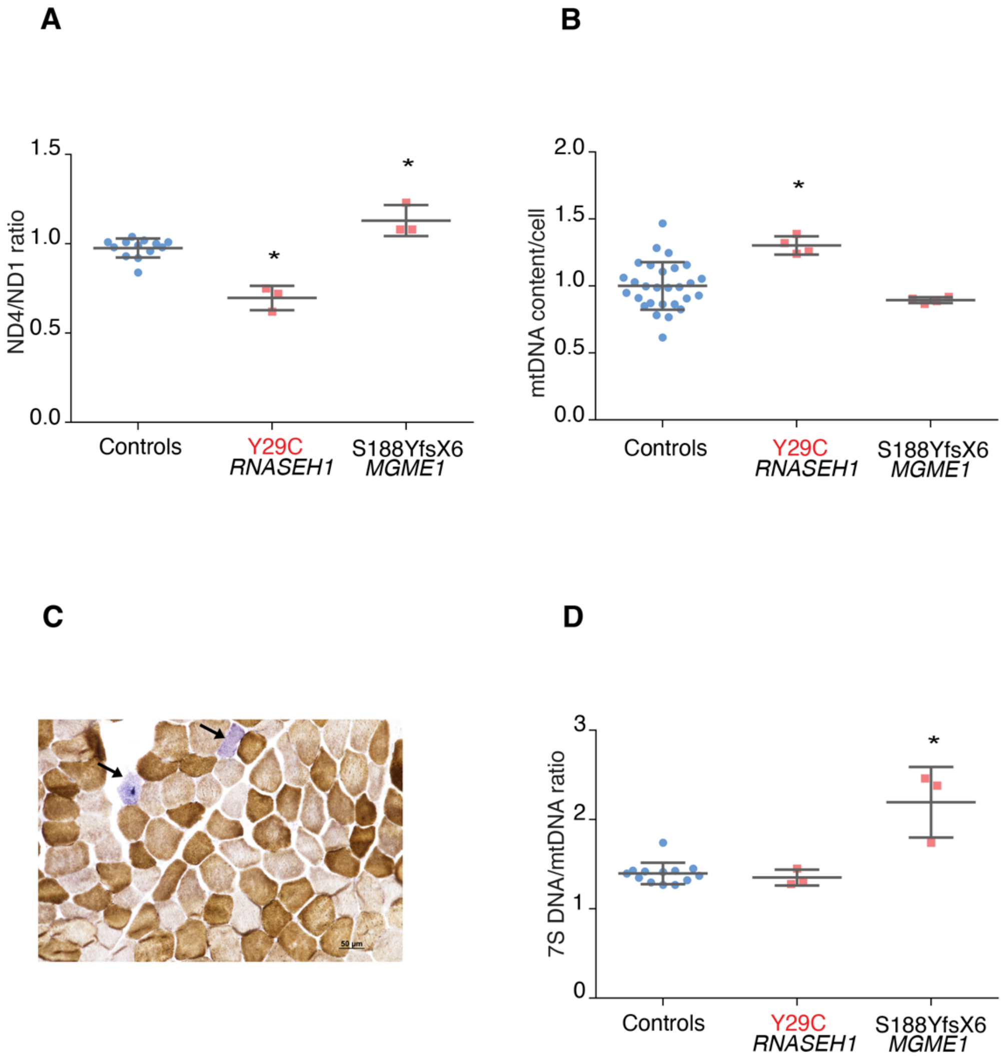

Fig. S4. mtDNA assessment in patient skeletal-muscle biopsy specimens. Quantification of mtDNA deletions (**A**) and mtDNA copy number **(B)** in skeletal muscle biopsies of healthy individuals (Controls) and Y29C (*RNASEH1)* and S188YfsX6 (*MGME1)* patients. Data are shown as scatter plot with mean (± SD). Amount of mtDNA deletions is expressed as percentage of ND4 (major arc, commonly deleted) on ND1 (minor arc, commonly not deleted). One-way ANOVA with Dunnett's multiple comparisons test, $p<0.05$. (**C**), COX/SDH staining of skeletal muscle from the S188YfsX6 (*MGME1)* patient. A few COX-negative fibers are seen as indicated by black arrows. Magnification 20x, scale bar 50 µm. **(D)**, Quantification of 7S DNA in skeletal muscle biopsies of healthy individuals (Controls) and Y29C (*RNASEH1)* and S188YfsX6 (*MGME1)*

patients. Data are shown as scatter plot with mean (± SD). One-way ANOVA with Dunnett's multiple comparisons test, $p<0.05$.

Table S1. Key reagents and resources used in this study.

**Reagent**	**Source**	**Identifier**
Amersham ECL Western Blotting Detection Reagent	GE Healthcare	Cat# RPN2106
anti-AIF	Abcam	Cat# ab16501
anti-GAPDH	Abcam	Cat# ab8245
anti-Histone 3	Sigma Aldrich	Cat# H0164
anti-HSP60	Enzo Life	Cat# ADI-SPA-807-E
anti-LRPPRC	Sigma Aldrich	Cat# HPA 036409
anti-mouse IgG horseradish peroxidase	GE Healthcare	Cat# NA9310V
anti-OXPHOS human	Abcam	Cat# ab110411
anti-OXPHOS rodent	Abcam	Cat# ab110413
anti-rabbit IgG horseradish peroxidase	GE Healthcare	Cat# NA9340V
anti-RNase H1	Proteintech	Cat# 15606-1-AP
anti-TFAM	Abcam	Cat# ab131607
anti-TIM 22	Proteintech	Cat# 14927-1-AP
anti-TOM 20	Cell Signalling	Cat# 13929S
anti-VDAC	Abcam	Cat# ab14734
BamHI	New England Biolabs	Cat# R0136S
BclI	New England Biolabs	Cat# R0160S
Digitonin	Merck	Cat# 300410
DMEM GlutaMAX	Thermo Fisher Scientific	Cat# 31966021
EcoRI	New England Biolabs	Cat# R0101S
Gibco Fetal Bovine Serum	Thermo Fisher Scientific	Cat# 10500064
Gibco Penicilin/Streptomycin	Thermo Fisher Scientific	Cat# 15-140-122
Iodonitrotetrazolium chloride	Merck	Cat# I10406
PerfectHyb Plus Hybridization buffer	Merck	Cat# H7033
Purified anti-POLRMT	N.-G.Larsson	Kuhl et al, 2014

Rapid hyb Buffer	Cytiva	Cat# RPN1635
SacI-HF	New England Biolabs	Cat# R3156S
XhoI	New Englands Biolabs	R0146S
EagI-HF	New Englands Biolabs	R3505S
BclII	New Englands Biolabs	R0144S
Triton X-100	Merck	Cat# T8787
Trizol reagent	Thermo Fisher Scientific	Cat#15596026
T4 Polynucleotide Kinase	New England Biolabs	Cat# M0201
RNase H	New England Biolabs	Cat# M0297S
Human PolG A&B	Posse et al, 2019	N/A
Human RNase H1 and mutants	Al-Behadili et al, 2018, this work	N/A
Human POLRMT	Posse et al, 2019	N/A
Human TFB2M	Posse et al, 2019	N/A
Human mtSSB	Posse et al, 2019	N/A
Human TFAM	Posse et al, 2019	N/A
**Critical Commercial Assays**		
Cell Fractionation Kit	Abcam	Cat# ab109719
Gentra Puregene Tissue Kit	Qiagen	Cat# 158667)
High-Capacity cDNA Reverse Transcription Kit	Thermo Fisher Scientific	Cat# 4368813
miRNeasy Mini Kit	Qiagen	Cat# 217004
Prime-It II Random Primer Labeling Kit	Agilent	Cat# 300385
TaqMan™ Universal Master Mix II, with UNG	Thermo Fisher Scientific	Cat# 4440038
TURBO DNA-free™ Kit	Thermo Fisher Scientific	Cat# AM1907
**Experimental Models: Organisms/Strains**		
Mouse: C57BL/6N	Charles River	N/A
Mouse: *Ckmm Cre*	The Jackson Laboratory	N/A
Mouse: *Rnaseh1* $^{+/-}$	This work	N/A
Mouse: *Rnaseh1*$^{loxP/loxP}$	TACONIC	Custom order
Mouse: *Mgme1* $^{-/-}$	N.-G. Larsson	Matic et al, 2018
Mouse: *Tefm* $^{loxP/loxP, +/Ckmm-cre}$	N.-G. Larsson	Jiang et al, 2019

**Primers**		
CACGACCAAGTGACAGCAAT	Sigma-Aldrich	Cre-FW (genotyping)
AGAGACGGAAATCCATCGCT	Sigma-Aldrich	Cre-RV (genotyping)
CCTAGGATTGGGAGTTTAAGGC	Sigma-Aldrich	*Rnaseh1* primer 29 FW (genotyping)
TCTAATGTCCCTTAGAGTAGGCTTC	Sigma-Aldrich	*Rnaseh1* primer 30 RV (genotyping)
GCAACCTGCACATAGATTCG	Sigma-Aldrich	*Rnaseh1* primer 25 FW(genotyping)
TTGCGGGCTCGGGATGTTC	Sigma-Aldrich	*Rnaseh1* exon 1 FW (RT PCR)
CCTTGCTCACTACAGCCGAG	Sigma-Aldrich	*Rnaseh1* exon 3 RV (RT PCR)
GACATATAATATTAACTATCA AACCCTATGTCCTGATCAATTCTA	Sigma-Aldrich	7S RNA primer (northern blot)
ATCAATGGTTCAGGTCATAA AATAATCATCAAC	Sigma-Aldrich	O_H FW (Southern blot probe 2DNAGE)
GCCTTAGGTGATTGGGTTTTGC	Sigma-Aldrich	O_H RV (Southern blot probe 2DNAGE)
TGACTTGTCCCACTAATAATCGGAG	Sigma-Aldrich	O_L FW (Southern blot probe 2DNAGE)
CCCAAAGAATCAGAACAGATGCTG	Sigma-Aldrich	O_L RV (Southern blot probe 2DNAGE)
ARC140: /5AmMC6/ACACTCTTTCC CTACACGACGCTCTTCCGATCT		HydEn seq primer
ARC76: GTGACTGGAGTTCAGAC GTGTGCTCTTCCGATCTNNNN*N*N		HydEn seq primer
ARC77: AGATCGGAAGAGCACAC GTCTGAACTCCAGTC*A*C		HydEn seq primer
ARC78CAAGCAGAAGACGGCATAC GAGATCGTGATGTGACTGGAGTTC AGACGTGTGCTCTTCCGATCT		HydEn seq primer
ARC84 CAAGCAGAAGACGGCATA CGAGATACATCGGTGACTGGAGTT CAGACGTGTGCTCTTCCGATCT		HydEn seq primer
ARC85 CAAGCAGAAGACGGCATAC GAGATGCCTAAGTGACTGGAGTTC AGACGTGTGCTCTTCCGATCT		HydEn seq primer
ARC86 CAAGCAGAAGACGGCATACG AGATTGGTCAGTGACTGGAGTTCAG ACGTGTGCTCTTCCGATCT		HydEn seq primer
ARC87 CAAGCAGAAGACGGCATAC GAGATCACTGTGTGACTGGAGTTCA GACGTGTGCTCTTCCGATCT		HydEn seq primer

ARC88 CAAGCAGAAGACGGCATACG AGATATTGGCGTGACTGGAGTTCAG ACGTGTGCTCTTCCGATCT		HydEn seq primer
ARC89 CAAGCAGAAGACGGCATACG AGATGATCTGGTGACTGGAGTTCAG ACGTGTGCTCTTCCGATCT		HydEn seq primer
ARC90 CAAGCAGAAGACGGCATACG AGATTCAAGTGTGACTGGAGTTCAG ACGTGTGCTCTTCCGATCT		HydEn seq primer
ARC91 CAAGCAGAAGACGGCATACG AGATCTGATCGTGACTGGAGTTCAG ACGTGTGCTCTTCCGATCT		HydEn seq primer
ARC93 CAAGCAGAAGACGGCATACG AGATAAGCTAGTGACTGGAGTTCAG ACGTGTGCTCTTCCGATCT		HydEn seq primer
ARC94 CAAGCAGAAGACGGCATACG AGATGTAGCCGTGACTGGAGTTCAG ACGTGTGCTCTTCCGATCT		HydEn seq primer
ARC95 CAAGCAGAAGACGGCATACG AGATTACAAGGTGACTGGAGTTCAG ACGTGTGCTCTTCCGATCT		HydEn seq primer
ARC160 AATGATACGGCGACCACCG AGATCTACACCTTCGCCTACACTCTT CCCTACACGACGCTCTTCCGATC		HydEn seq primer
**Taqman Assays and Oligonucleotides**		
m-12S	Thermo Fisher Scientific	Mm04260177_s1
m-16S	Thermo Fisher Scientific	Mm04260181_s1
m-18S	Thermo Fisher Scientific	Mm03928990_g1
m-Actin	Thermo Fisher Scientific	Mm01205647_g1
m-Atp6	Thermo Fisher Scientific	Mm03649417_g1
m-Atp8	Thermo Fisher Scientific	Mm04225236_g1
m-Cox1	Thermo Fisher Scientific	Mm04225243_g1
m-Cox2	Thermo Fisher Scientific	Mm03294838_g1
m-Cox3	Thermo Fisher Scientific	Mm04225261_g1
m-Cyb	Thermo Fisher Scientific	Mm04225271_g1
m-Nd1	Thermo Fisher Scientific	Mm04225274_g1
m-Nd2	Thermo Fisher Scientific	Mm04225288_s1
m-Nd3	Thermo Fisher Scientific	Mm04225292_g1

m-Nd4l/4	Thermo Fisher Scientific	Mm04225294_s1
m-Nd5	Thermo Fisher Scientific	Custom made_AIHSNT9
m-Nd6	Thermo Fisher Scientific	Custom made_AIVI3E8
m-Rnaseh1	Thermo Fisher Scientific	Mm00488036_m1
**Recombinant DNA and Oligonucleotides for in vitro assays**		
GCAAATTCGAAGAAGCAGCTTCAAACCT GCCGGGGCTTCTCCCGCCTTTTTCCTAA TAATAAAGAGGACAGATTGAAGC	Eurofins	80 mer template for RNA:DNA hybrids
LSP template (nt 1-477 of mtDNA): pUC18 DNA with mtDNA seq ligated between BamHI and HindIII	Posse et al, 2019	N/A
AAAAAAGGCGGGAGAAGCCCCG GCAGGTTTGAAGCTGCTTCTTCGAATTTGC	Eurofins	26DNA:26 RNA for RNA:DNA hybrids
**Probes**		
pAM1 /mouse mtDNA cloned in pAcyc177	Gift from D.Clayton	N/A
mouse 7S DNA for Southern Blot	Matic et al, 2018	N/A
TCCCAAGATCCAACTACGAGCTTTTTAAC TGCAGCAACTTTAATATACGCTATTGGAG CTGGAATTACCGCGGCTGCTGGCACCAGA CTTGCCCTCCAATGGATCCTCGTTAAAGG ATTTAAAGTGGACTCATTCCAATTACAGG GCCTCGAAAGAGTCCTGTATTGTTATTTT CGTCACTACCTCCCCGGGTCGGGAGTGGG TAATTTGCGCGCCTGCTGCCTTCCTTGGAT GTGGTAGCCGTTTCTCAGGCTCCCTCTCCG GAATCGAACCCTGATTCCCCGTCACCCGT GGTCACCATGGTAGGCACGGCGACTACC ATCGAAAGTTGATAGGGCAGACGTTCGA ATGGGTCGTCGCCGCCACGGGGGGCGTG CGATCGGCCCGAGGTTATCTAGAGTCACC AAAGCCGCCGGCGCCCGACCCCCGGCCG GAGCCGGGAGGGAGCTCACCGGGTTGGTT TTGATCTGATAAATGCACGCATCCCCCCC GGGAAGGGGGGTCAGCGCCCGTCGGCATG TATTAGCTCTAGAATTACCACAGTTATCCA AGTAGGAGAGGAGCGAGCGACCAAAGGA ACCATAACTGATTTAATGAGCCATTCGCA GTTTCACTGTACCGGCCGTG	N/A	mouse 18S probe for Southern blot
mouse 12S probe for Southern blot	Jiang et al, 2019	N/A

mouse 5.8S probe for Northern blot	Jiang et al, 2019	N/A
human 18S probe for Southern blot	Nicholls et al, 2014	N/A
TTACAGTCAAATCCCTTCTCGTCCCCAT GGATGACCCCCCTCAGATAGGGGTCCC TTGACCACCATCCTCCGTGAAATCAATA TCCCGCACAAGAGTGCTACTCTCCTCGC TCCGGGCCCATAACACTTGGGGGTAGCT AAAGTGAACTGTATCCGACATCTGGTTC CTACTTCAGGGTCATAAAGCCTAAATAG CCCACACGTTCCCCTTAAATAAGACATC ACGATG	N/A	human 7SDNA for Southern blot
